# The Fusaric Acid Derivative qy17 Inhibits *Staphylococcus haemolyticus* by Disrupting Biofilm Formation and the Stress Response *via* Altered Gene Expression

**DOI:** 10.3389/fmicb.2022.822148

**Published:** 2022-03-14

**Authors:** Bing Wang, Chao-Rong Song, Qing-Yan Zhang, Peng-Wei Wei, Xu Wang, Yao-Hang Long, Yong-Xin Yang, Shang-Gao Liao, Hong-Mei Liu, Guo-Bo Xu

**Affiliations:** ^1^Engineering Research Center of Medical Biotechnology & School of Basic Medical Sciences, Guizhou Medical University, Guiyang, China; ^2^Key Laboratory of Infectious Immune and Antibody Engineering in Guizhou Province, Guiyang, China; ^3^School of Biology and Engineering, Guizhou Medical University, Guiyang, China; ^4^State Key Laboratory of Functions and Applications of Medicinal Plants, Guizhou Medical University, Guiyang, China; ^5^Key Laboratory of Environmental Pollution Monitoring and Disease Control, China Ministry of Education (Guizhou Medical University), Guiyang, China; ^6^School of Pharmacy, Guizhou Medical University, Guiyang, China

**Keywords:** *Staphylococcus haemolyticus*, antimicrobial activity, biofilm, fusaric acid analogs, toxins

## Abstract

*Staphylococcus haemolyticus (S. haemolyticus)* is the second most commonly isolated coagulase-negative *staphylococcus* (CoNS) in patients with hospital-acquired infections. It can produce phenol-soluble modulin (PSM) toxins and form biofilms. Compared with the wealth of information on *Staphylococcus aureus* and *Staphylococcus epidermidis*, very little is known about *S. haemolyticus*. There is an urgent need to find an effective preparation to combat the harm caused by *S. haemolyticus* infection. Chinese herbs have been utilized to cure inflammation and infectious diseases and have a long history of anticancer function in China. Here, we modified fusaric acid characterized from the metabolites of *Gibberella intermedia*, an endophyte previously isolated from *Polygonum capitatum*. This study shows that fusaric acid analogs (qy17 and qy20) have strong antibacterial activity against *S. haemolyticus*. In addition, crystal violet analyses and scanning electron microscopy observations demonstrated that qy17 inhibited biofilm formation and disrupted mature biofilms of S. *haemolyticus* in a dose-dependent manner. Additionally, it reduced the number of live bacteria inside the biofilm. Furthermore, the antibiofilm function of qy17 was achieved by downregulating transcription factors (*sigB*), transpeptidase genes (*srtA*), and bacterial surface proteins (*ebp*, *fbp*) and upregulating biofilm-related genes and the density-sensing system (*agrB*). To further elucidate the bacteriostatic mechanism, transcriptomic analysis was carried out. The following antibacterial mechanisms were uncovered: (i) the inhibition of heat shock (*clpB*, *groES*, *groL*, *grpE*, *dnaK*, *dnaJ*)-, oxidative stress (*aphC*)- and biotin response (*bioB*)-related gene expression, which resulted in *S. haemolyticus* being unable to compensate for various stress conditions, thereby affecting bacterial growth; and (ii) a reduction in the expression of PSM-beta (PSMβ1, PSMβ2, PSMβ3) toxin- and Clp protease (*clpP*, *clpX*)-related genes. These findings could have major implications for the treatment of diseases caused by *S. haemolyticus* infections. Our research reveals for the first time that fusaric acid derivatives inhibit the expression of biofilm formation-related effector and virulence genes of *S. haemolyticus*. These findings provide new potential drug candidates for hospital-acquired infections caused by *S. haemolyticus.*

## Introduction

Coagulase-negative *staphylococci* (CoNSs) are important nosocomial pathogens. Among CoNSs, *Staphylococcus haemolyticus* was isolated from human blood cultures with the second highest frequency, slightly less than that of *S. epidermidis* (Farina et al., 2013; Becker et al., 2020). *S. haemolyticus* has been reported to be associated with congenital valvular endocarditis, sepsis, peritonitis, and urinary tract, wound and bone and joint infections (Falcone et al., 2007; Farina et al., 2013; Grant and Hung, 2013; Yu et al., 2017; Heilmann et al., 2019). Studies have found that there are some toxins produced by CoNSs that promote or trigger sepsis (Barros et al., 2012; Farina et al., 2013; Qin et al., 2017; Becker et al., 2020), which is a serious blood infection and the most common cause of death in hospitalized patients (Dellinger et al., 2008). For example, phenol-soluble modulins (PSMs), including PSMα, PSMβ1, PSMβ2, and PSMβ3, are toxins that cause hemolysis, which contributes directly to the virulence of *S. haemolyticus* (Cheung et al., 2014; Da et al., 2017; Xu et al., 2017). It is estimated that approximately 1 million people in the United States suffer from sepsis each year, 28–50% of these cases are fatal, and *S. haemolyticus* is considered one of the most common causes (Jawad et al., 2012).

Infections caused by CoNSs most often occur after medical device implantation and are attributed to the potential biofilm formation of CoNSs. Clinically, medical device infections and chronic infections, including catheter, pacemaker, and prosthetic joint infections (Grant and Hung, 2013; Jiang et al., 2019; Becker et al., 2020), are associated with the formation of biofilms (Francolini and Donelli, 2010). Bacterial biofilms are formed with extracellular polymeric substances (EPS) and even host substances, including polysaccharides, proteins, lipids and extracellular DNA (eDNA) (Karygianni et al., 2020). Once biofilms form, it is difficult for the host immune system to eliminate the bacteria encased in the biofilm, and biofilms have a inhibitory effect on phagocytosis by macrophages in the body (Dragos and Kovacs, 2017). Additionally, the bacteria in the biofilm lack nutrition and exhibit slow growth and low metabolic activity, which allows the bacteria in the biofilm to easily persist for a long time and develop resistance to antibiotics (Grant and Hung, 2013; Koo et al., 2017; Becker et al., 2020; Mishra et al., 2020). High heterogeneity of bacterial biofilms, including chemical, bacterial physiological, and bacterial genetic heterogeneity, leads to different complex structures, channels, variable oxygen ion concentrations and differential subclones (Stewart and Franklin, 2008). Furthermore, the metabolic states of the bacteria are quite different from the surface to the interior of the biofilm. This biofilm environment results in the production of different metabolites and altered the pH and osmotic pressure. In combination with genetically heterogeneous subclones, an increasing number of extensively drug-resistant bacteria have evolved (Stewart and Franklin, 2008; Ciofu and Tolker-Nielsen, 2019; Podlesek and Zgur Bertok, 2020). Thus, biofilms can trap bacteria, making them harder to eliminate. In addition, biofilms act as a reservoir of bacterial cells that can cause new infection of the patient when the biofilm matures. *S. haemolyticus* plays an important role in hospital-acquired opportunistic infections associated with implanted medical devices (Mack et al., 2006; Barros et al., 2012; Grant and Hung, 2013; Becker et al., 2020). Furthermore, among all CoNS species, *S. haemolyticus* has the highest level of drug resistance and is resistant to glycopeptides and β-lactams (Tabe et al., 2001; Szczuka et al., 2015). This limits the available treatment options and makes *S. haemolyticus* infections a serious threat. Therefore, there is an urgent need to identify effective antibiofilm preparations to prevent the harm caused by biofilm-related infections.

Endophytes from medicinal plants are abundant resources and have value for research (Gomez and Luiz, 2018; Mishra et al., 2020). Their metabolites provide a rich source of natural products for drug discovery (Gomez and Luiz, 2018). Many reports have confirmed that endophyte metabolites have biological functions such as antitumor (El-Sayed et al., 2021), antiviral (Peng et al., 2020), and antibacterial (Thanh et al., 2020) activities. Searching for antibacterial ingredients derived from Chinese herbal endophytes provides a new approach for the research and development of new antibacterial drugs. Their wide availability and low cost are also advantageous for further development of this approach. In a previous report, we isolated an endophytic fungus (*Gibberella intermedia*) from *Polygonum capitatum*, a traditional Chinese herb that has a long history of use in treating urinary tract infections, and analyzed its metabolites (Zhang et al., 2021).

Fusaric acid (FA), 5-butylpicolinic acid, is a mycotoxin that has been isolated from certain species of *Fusarium* and is a kind of *Fusarium* natural fungal metabolite (Bacon et al., 1995; Thanh et al., 2020). Studies have shown that FA has good antibacterial effects (Huang et al., 2020). However, the antibacterial effect and molecular mechanism of FA against *S. haemolyticus* are still unclear. Therefore, based on the previous extraction of FA from the endophytic fungus *Gibberella intermedia* from *Polygonum capitatum*, followed by its use as a template for modification and transformation, 42 derivatives were obtained, including 5-(4-butylphenyl) picolinic acid (qy17) and 5-(4-(tert-butyl) phenyl) picolinic acid (qy20) (Zhang et al., 2021). Here, the antibacterial function and lineage of qy17 and qy20 were characterized. The antibiofilm function of qy17 was also demonstrated. This study explores the potential mechanism of the activity of the derivative qy17 against *S. haemolyticus* and provides new drug candidates and a theoretical basis for clinical treatment.

## Materials and Methods

### Synthesis of Fusaric Acid Analogs

**FIGURE 1:**
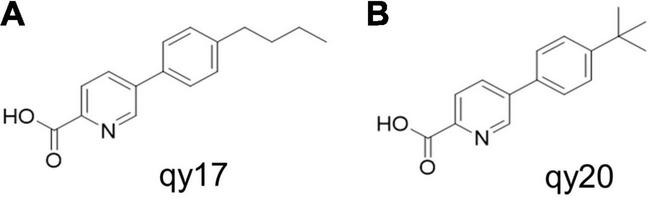
Antimicrobial activity of fusaric acid derivatives. **(A)** qy17 molecular structure. **(B)** qy20 molecular structure.

The FA analogs 5-(4-butylphenyl) picolinic acid (qy17, Figure 1A) and 5-(4-(tert-butyl) phenyl) picolinic acid (qy20, Figure 1B) were synthesized *via* the Suzuki coupling reaction and ester hydrolysis reaction from the starting material methyl 5-bromopicolinate according to previously reported methods. In our preliminary experiments, the data showed that qy17 had a better antibacterial effect against *Enterococcus faecium* than qy20. Moreover, it had better solubility and was more stable than qy20 in a minimal inhibitory concentration (MIC) test and time-growth curve assay. Thus, qy17 was chosen for transcriptomic analysis and subsequent experiments.

### Bacterial Strains and Culture Conditions

Bacterial strains (*S. haemolyticus, S. epidermidis, E. faecium, Pseudomonas aeruginosa*, and *Klebsiella pneumoniae* clinical strains) were provided by Shan Wan, deputy chief physician of the First Affiliated Hospital of Guizhou Medical University, and were identified using the Microbiological Immunology Automatic Bacterial Identification and Drug Sensitivity Analysis System (WalkAway 96) of the First Affiliated Hospital of Guizhou Medical University. *S. haemolyticus* ATCC 29970 and *Escherichia coli* ATCC R25922 were ordered from ATCC. These strains were cultured overnight in tryptic soy broth (TSB; Sangon Biotech, China) at 37°C and 200 rpm. The bacterial seed solution was diluted to 10^7^ CFUs/mL. Then, 100 μL of diluent was added to the detection medium (1:2) for 2 gradient dilutions, and the working inoculum concentration was 10^6^ CFUs/mL (Wang et al., 2021a,b).

### Antibacterial Activity Test

The MIC and minimum bactericidal concentration (MBC) were determined by the microbroth dilution method (Wang et al., 2021a,b). A single colony was placed in TSB medium and cultured overnight. Qy17 and qy20 were prepared at a concentration of 2–256 μg/mL in sterile Mueller-Hinton broth (MHB) medium. Then, 100 μL of working bacterial solution was added to each well, and the final total volume of each well was 200 μL (final bacterial concentration: 10^6^ CFUs/mL). The samples were incubated on a shaker at 150 rpm at 37°C for 20 h. The MIC and MBC were visually inspected, and TSB agar plate diffusion verification was performed.

### Time-Growth Curve of Bacteria

The kinetics of the bactericidal action of qy17 and qy20 on *S. haemolyticus* were studied with an improved method (Yu et al., 2020). A 96-well plate contained a bacterial suspension (5 × 10^7^ CFUs/mL, final concentration) and MHB containing qy17 and qy20 (8, 16, and 32 μg/mL, final concentrations). The plate was incubated at 37°C, with DMSO (content less than 2.56%) included as the solvent control. Then, a microplate reader was used to measure the OD_595nm_ value of the samples at different time points (0, 4, 8, 12, and 24 h). Time was used as the abscissa, and the OD_595nm_ value was used as the ordinate to generate a bacterial growth curve.

### *Staphylococcus haemolyticus* Biofilm Inhibition Assay

Preparation of *S. haemolyticus* biofilms in 96-well polystyrene microplates was performed according to previously reported methods (Wang et al., 2021a,b). Briefly, *S. haemolyticus* was grown overnight in TSB at 37°C and 200 rpm. After washing twice with 0.9% saline and diluting to approximately 10^7^ CFUs/mL, 100 μL of logarithmic-phase *S. haemolyticus* was inoculated into 100 μL of TSB (1% glucose) solution containing different concentrations of qy17 (8, 16, and 32 μg/mL) or qy20 (16, 32, and 48 μg/mL). DMSO was used as the solvent control, TSB with 1% glucose was used as the negative control, and the cells were incubated at 37°C for 24 h. Planktonic bacteria were carefully discarded, and then the samples were washed twice gently with PBS and fixed with 4% paraformaldehyde for 20 min. The paraformaldehyde was discarded, and the biofilms were stained with 0.1% crystal violet at room temperature for 20 min, followed by washing with 0.9% saline to remove unbound stain. The 96-well plates were dried, and a Cytation 5 was used to capture images at different magnifications. Then, 30% glacial acetic acid was added to each well to dissolve the stain, and the absorbance of the crystal violet solution was read at 492 nm. The amount of biofilm was directly proportional to the OD value of the crystal violet solution.

### Mature *Staphylococcus haemolyticus* Biofilm Disruption Assay

As described above, mature *S. haemolyticus* biofilms were preformed in a 96-well polystyrene microplate after 24 h of incubation. Briefly, *S. haemolyticus* was incubated in TSB with 1% glucose at 37°C for 24 h. Then, qy17 was added to the well for another 24 h of culture. Planktonic bacteria were removed, 200 μL of fresh TSB with 1% glucose with different concentrations of qy17 (32, 48, and 64 μg/mL) or qy20 (32, 48, and 64 μg/mL) was added to each well, and the plate was incubated for another 24 h at 37°C; DMSO was included as the solvent control, and TSB with 1% glucose was included as the negative control. Finally, the abovementioned 0.1% crystal violet staining method was used to analyze the damaging effect of qy17 on mature biofilms. A Cytation 5 was utilized to capture images at different magnifications as mentioned above. Then, 30% glacial acetic acid was utilized to dissolve the stain, and biofilms were quantified as mentioned above.

### Scanning Electron Microscopy Assay

*S. haemolyticus* was seeded into 6-well plates with cover glass slips. A total of 100 μL of TSB with 1% glucose containing 16 μg/mL qy17 was added, and the plates were incubated at 37°C for 24 h. After incubation, the supernatant was gently discarded, and the samples were washed twice gently with 0.9% saline. Then, 2.5% glutaraldehyde at 4°C was used for fixation for 24 h at room temperature. As described above, biofilms were formed in a 6-well polystyrene microplate for 24 h. The supernatants were discarded, 200 μL of fresh TSB with 1% glucose without or with 64 μg/mL qy17 was added to the biofilms, and the samples were incubated for another 24 h. The supernatant was then gently discarded, and the cells were gently washed twice with 0.9% saline. Finally, the samples were fixed with 2.5% glutaraldehyde at 4°C for 24 h. Scanning electron microscopy (SEM) inspection was carried out as described previously.

### Analysis of *Staphylococcus haemolyticus* Viability Within Biofilms by the MTT and Colony-Forming Unit (CFU) Methods

Bacterial viability was analyzed using the 3-(4,5-dimethylthiazol-2-yl)-2,5-diphenyl tetrazolium bromide (MTT, Sigma–Aldrich, United States) protocol. As mentioned above, for the biofilm formation process, 8, 16, and 24 μg/mL qy17 were used as previously described. For preformed 24-h mature biofilms, qy17 was used at 32, 48, and 64 μg/mL. The supernatant was removed, and the biofilm was retained at the bottom of the well. MTT (0.5 mg/mL) was used to detect live bacteria in the biofilms of the control group and in the groups treated with different concentrations of qy17. For the Colony-Forming Unit (CFU) method, the biofilm was resuspended in 0.9% normal saline and mixed well, and then TSB agar plates were used for colony count analysis.

### Bacterial Total RNA Extraction and Real-Time PCR Analysis

After *S. haemolyticus* was grown in TSB with 1% glucose for 3 h, DMSO and qy17 were added to the solvent and drug groups, respectively. After culturing for another 21 h, the cells were centrifuged at 1,2000 rpm for 2 min to collect all bacteria. DEPC-treated water was used for initial washes. A Tissue RNA Purification Kit Plus (ES Science) was used to isolate RNA. Total RNA was reverse transcribed into cDNA template using a Prime Script™ RT Reagent Kit with gDNA Eraser (Shang Hai Yi Shan Biotechnology Co., Ltd.). Real-time PCR (RT–PCR) was utilized to detect gene expression. 16S rRNA was selected as the internal reference gene. A 2 × TG qPCR Master Mix Kit (TAKARA) and a StepOnePlus Real-Time PCR system (Applied Biosystems, CA, United States) were used for RT–PCR. The program settings were as follows: 95°C for 30 s, followed by 95°C for 10 s, 55°C for 30 s, and 72°C for 30 s for a total of 40 cycles. The primers used in this study are listed in Supplementary Table 1.

### Total RNA Extraction for Transcriptome Sequencing

Three *S. haemolyticus* culture groups were included: a solvent control group (without qy17) and treatment groups supplemented with a subinhibitory concentration (MIC-8 μg/mL) of qy17 and an inhibitory concentration (MIC-16 μg/mL). The cultures were placed in a shaker at 37°C and 150 rpm to grow for 18 h, and then total bacteria were collected for RNA extraction. rRNA was removed, possible DNA contamination was digested with DNase I, and fragmentation buffer was added to break the RNA into short fragments. Using fragmented RNA as a template, six-base random hexamers were used to synthesize first-strand cDNA, and then buffer, dNTPs and DNA polymerase I were added to synthesize double-stranded cDNA. The purified double-stranded cDNA was then repaired, tails were added, and sequencing adaptors were attached. After the adaptors were added, the samples were treated with UDGase to degrade double-stranded cDNA containing uracil. Then, AMPure XP beads were used for fragment size selection, PCR enrichment was performed, and the final cDNA library was collected. After the library was constructed, a Qubit 2.0 was used for preliminary quantification. The library was then diluted to 1 ng/μL, and an Agilent 2100 was used to detect the insert size of the library. Once the insert size met expectations, RT–PCR was used to determine the effective concentration of the library (Hua et al., 2019). Accurate quantification (effective library concentration > 2 nM) was performed to ensure library quality. After the library was qualified, the different libraries were pooled according to the effective concentration and the target off-machine data volume for HiSeq/MiSeq sequencing (Vaishampayan et al., 2018). The transcriptome data were deposited in the NCBI SRA database with the BioProject accession number PRJNA799664.

### Transcriptome Result Verification

The transcriptome data were further verified by RT–PCR to determine their validity. Three *S. haemolyticus* culture groups were used: the solvent group control (without qy17) and the treatment groups supplemented with qy17 at 8 and 16 μg/mL. The cultures were placed in a shaker at 37°C and 150 rpm and grown for 18 h, and then the bacterial cells were collected for total RNA extraction. The above RNA was reverse transcribed into a cDNA template using a Prime Script™ RT Reagent Kit with gDNA Eraser (Shang Hai Yi Shan Biotechnology Co., Ltd.). RT–PCR was utilized to detect gene expression. PCR was performed as follows: 95°C for 30 s, followed by 95°C for 10 s, 60°C for 30 s, and 72°C for 30 s for a total of 40 cycles. After normalizing to the 16S rRNA bacterial housekeeping gene as the internal control, the change in mRNA expression level was calculated (Livak and Schmittgen, 2001; Gowrishankar et al., 2015). The primer sequences used are shown in Supplementary Table 2.

### Hemolysis Assay

Directly ordered commercial 10% rat red blood cells (RBCs) (Cat: BC-RBC-RAT005; No. 20211208; Nanjing BioChannel Biotechnology Co., Ltd.) were diluted with RPMI-1640. Then, 100 μL of RBC suspension and 100 μL of RPMI-1640 (final concentration of 64, 32, 24, and 16 μg/mL qy17) were added to a 96-well plate (final RBC concentration of 4%), with an RPMI-1640 and a 0.1% Triton X-100 (100% hemolysis)-treated erythrocyte suspension serving as the negative and positive control, respectively. After 10 h of incubation, the samples were centrifuged at 500 × g for 5 min. Then, 100 μL of the supernatant was collected, the absorbance at 570 nm (A570) of the supernatant was measured, and the hemolysis rate was calculated. S. *haemolyticus* was cultured overnight at 37°C and 200 rpm in TSB and diluted with RPMI-1640 medium (10^7^ CFUs/mL), 100 μL of bacterial solution (final concentrations of 32, 24, and 16 μg/mL qy17) and 100 μL of RBCs (final RBC concentration of 4%) in a 96-well plate. RBCs were treated with DMSO and 0.1% Triton X-100 (100% hemolysis) as negative and positive controls, respectively, and incubated for 10 h before centrifuging at 500 × g for 5 min. Then, 100 μL of the supernatant was collected, the absorbance was measured at 570 nm, and the hemolysis rate was calculated.

### Statistical Analysis

**FIGURE 2:**
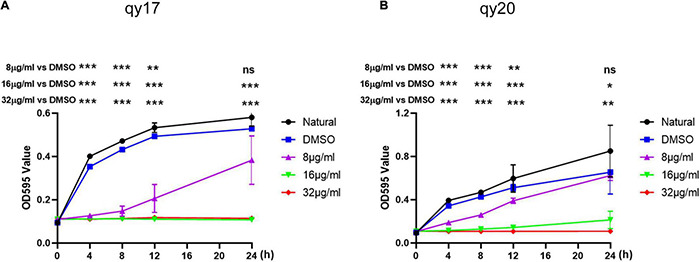
Time-growth curve of *S. haemolyticus* with qy17 and qy20. The strains were treated with different concentrations of qy17 and qy20 for 24 h, and the OD_595_ was measured with a microplate reader every 4 h. Growth curves of *S. haemolyticus* with qy17 **(A)** and qy20 **(B)** were drawn. Statistical analyses were determined with an unpaired *t*-test, and data are presented as the means ± standard deviations (*n* = 3). *P < 0.05, **P < 0.01, ***P < 0.001; ns: not significant, vs. DMSO. The * location corresponding to the following abscissa is the corresponding time point.

**FIGURE 3:**
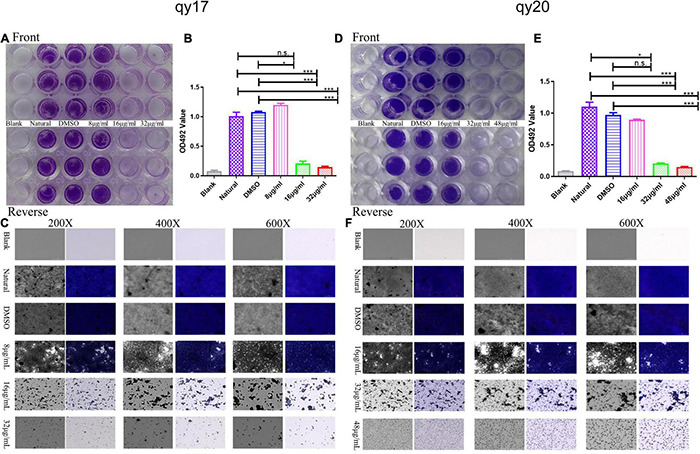
The effect of qy17 and qy20 on the nascent biofilm formation of *S. haemolyticus*. *S. haemolyticus* was treated with different concentrations of qy17 and qy20 for 24 h and stained with crystal violet to analyze the adhesion of bacterial biofilms to 96-well plates **(A,D)**. The biofilm was dissolved with glacial acetic acid, and the OD490 was measured **(B,E)**. A Cytation 5 was used to capture images at different magnifications **(C,F)**. Statistical analyses were determined with an unpaired test, and data are presented as the means ± standard deviations (*n* = 3). *P < 0.05, ***P < 0.001; ns: not significant vs. natural and DMSO.

**FIGURE 4:**
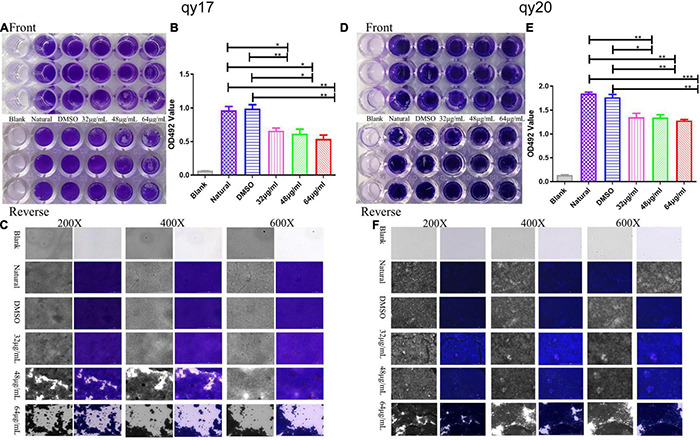
The effect of qy17 and qy20 on *S. haemolyticus* mature biofilms. Mature biofilms were preformed for 24 h. Then, the cells were treated with different concentrations of qy17 for 24 h. Crystal violet staining was used to analyze the adhesion of bacteria to 96-well plates **(A,D)**. The biofilm was dissolved with glacial acetic acid, and the OD490 was measured **(B,E)**. A Cytation 5 was used to capture images at different magnifications **(C,F)**. Statistical analyses were determined with an unpaired test, and data are presented as the means ± standard deviations (*n* = 3). *P < 0.05, **P < 0.01, ***P < 0.001; ns: not significant. vs. natural and DMSO.

**FIGURE 5:**
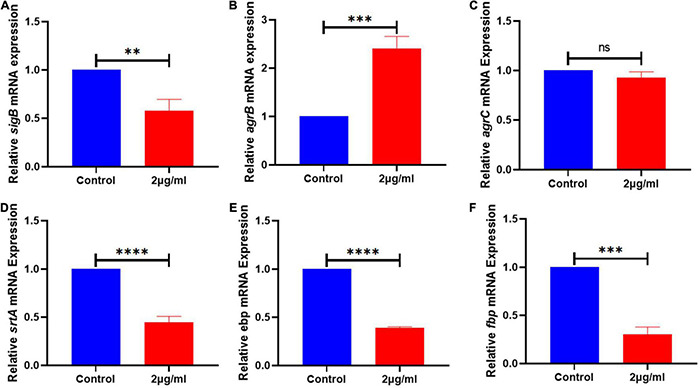
The expression of genes related to the biofilm signaling pathway of *S. haemolyticus* was altered by qy17. After 24 h of treatment with or without qy17, total bacterial RNA was isolated from *S. haemolyticus*, and real-time PCR was utilized to detect *sigB* **(A)**, *agrB* **(B)**, *agrC* **(C)**, *srtA* **(D)**, *ebp* **(E)**, and *fbp* **(F)** expression. Statistical analyses were determined with an unpaired *t*-test, and data are presented as the means ± standard deviations (*n* = 3). ^**^P < 0.01, ^***^P < 0.001, ****P < 0.0001; ns: not significant. vs. DMSO.

**FIGURE 6:**
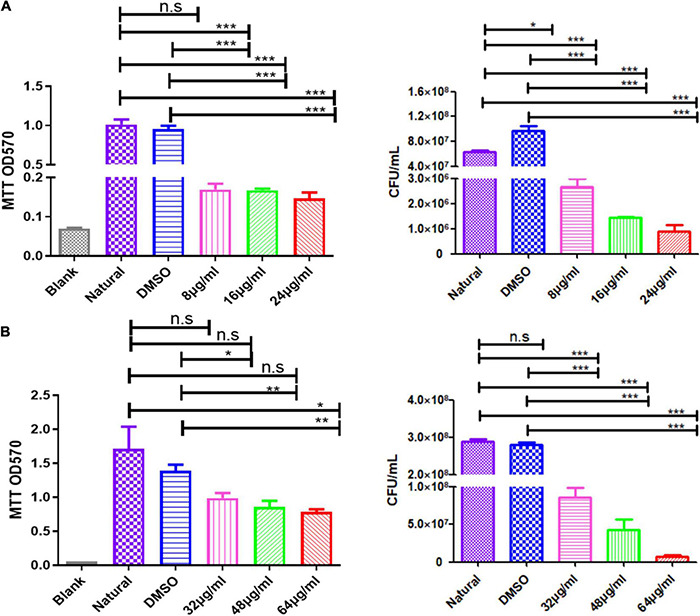
Qy17 reduces the number of live bacteria inside *S. haemolyticus* biofilms. The MTT method (left) and the colony-forming unit (CFU) counting method (right) were used to determine the number of viable cells in *S. haemolyticus* biofilms treated with qy17. **(A)** Biofilm formation. **(B)** Mature biofilms. Statistical analyses were determined with an unpaired *t*-test, and data are presented as the means ± standard deviations (*n* = 3). *P < 0.05, **P < 0.01, ***P < 0.001; ns: not significant. vs. natural and DMSO.

**FIGURE 7:**
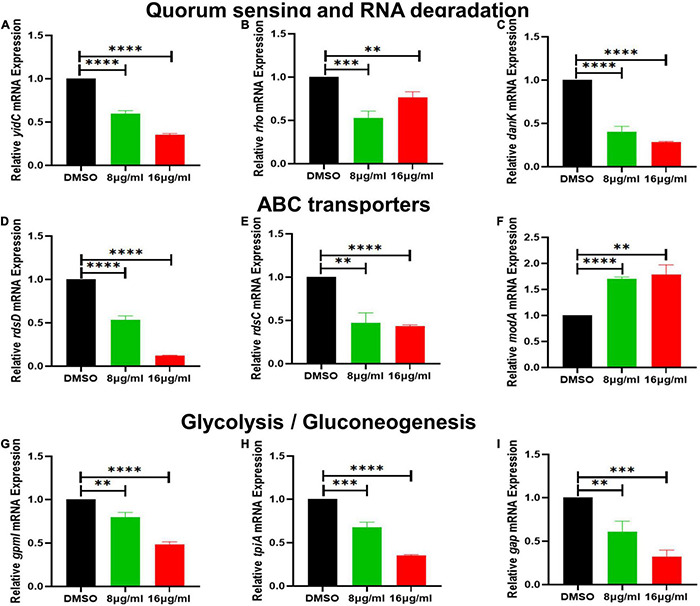
The effect of qy17 treatment on the gene expression of *S. haemolyticus* at different concentrations. **(A)** Quorum sensing gene expression (*yidC*). **(B,C)** RNA degradation gene expression (*rho*, *dnaK*). **(D–F)** ABC transporter gene expression (*rdsD*, *rdsC*, *modA*). **(G–I)** Glycolysis/gluconeogenesis gene expression (*gpmI*, *tpiA*, *gap*). Statistical analyses were determined with an unpaired *t*-test, and data are presented as the means ± standard deviations (*n* = 3). ^**^P < 0.01, ^***^P < 0.001, ^****^P < 0.0001, vs. DMSO.

**FIGURE 8:**
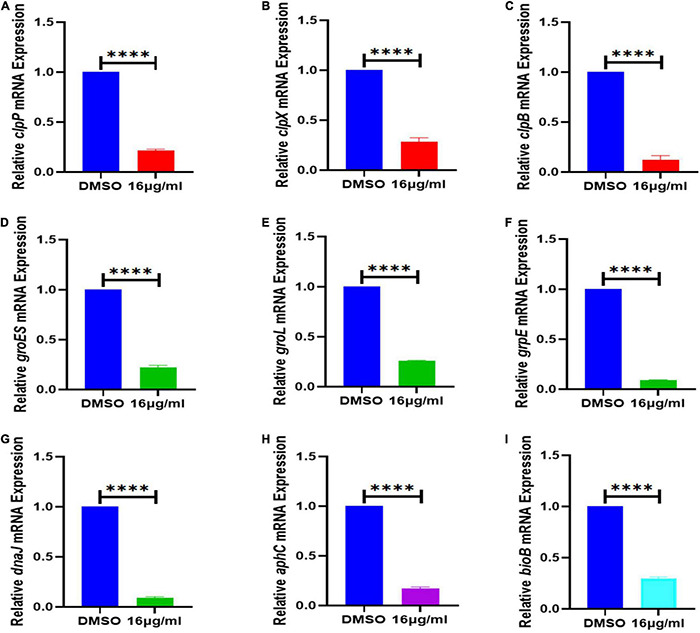
Effect of 16 μg/mL qy17 treatment on the gene expression of *S. haemolyticus*. This figure indicates that after *S. haemolyticus* was treated with qy17 for 18 h, the expression of **(A–C)** virulence-regulated genes (*clpP*, *clpX*, and *clpB*), **(D–H)** stress response genes (*groES*, *groL*, *grpE*, *dnaK*, *dnaJ*, and *aphC*) and **(I)** biotin synthase genes (*bioB*) decreased. Statistical analyses were determined with an unpaired *t*-test, and data are presented as the means ± standard deviations (*n* = 3). ^****^P < 0.0001, vs. DMSO.

**FIGURE 9:**
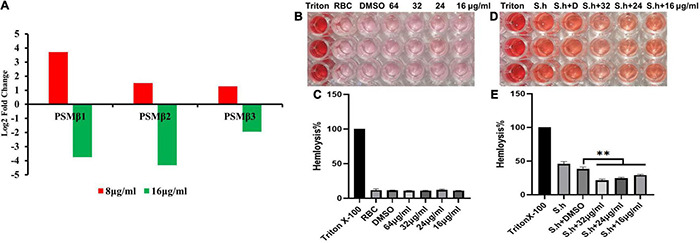
Qy17 reduces the expression of the PSMβ toxin gene and inhibits the hemolytic activity of *S. haemolyticus*. After treatment with different concentrations of qy17, the PSMβ (phenol-soluble modulin PSM-beta-1, PSM-beta-2, PSM-beta-3) toxin gene expression was determined at the transcriptional level. **(A)** PSMβ toxin gene expression was downregulated with 16 μg/mL qy17 treatment but upregulated after 8 μg/mL qy17 treatment. **(B,C)** Hemolysis assay of qy17. **(D,E)** Qy17 inhibits the hemolytic activity of *S. haemolyticus*. ^**^P < 0.01, vs. DMSO.

All experiments were performed at least three times and expressed as the mean ± standard deviation (SD). GraphPad Prism 8.3.0 was used to draw all figures. The results shown in Figure 2 were analyzed using the two-tailed Student’s *t*-test. The results shown in Figures 3–9 were analyzed using the two-tailed unpaired *t*-test. For all statistical analyses, a *p*-value less than 0.05 was considered statistically significant. *P*-values less than 0.001 were considered highly statistically significant.

## Results

### Analysis of the Antibacterial Activity of qy17 and qy20 Against *Staphylococcus haemolyticus*

For analysis of the antibacterial activity of qy17 and qy20, three gram-positive (G^+^) bacteria (*S. haemolyticus*, *S. epidermidis*, and *E. faecium*) and *K. pneumoniae* were utilized to conduct an antibacterial test to determine the MICs. The MICs of qy17 and qy20 against *S. epidermidis* and *S. haemolyticus* were 16 μg/mL, and the MICs against *E. faecium* were 8 μg/mL, while the compounds had almost no inhibitory effect on *K. pneumoniae* (Table 1). To further validate the antibacterial functions, a time-growth curve assay was performed for qy17 and qy20 with *S. haemolyticu*s. The antibacterial effects of qy17 and qy20 were enhanced with increasing concentrations in a concentration-dependent manner (Figure 2).

**TABLE 1:** Antimicrobial activities of qy17 and qy20.

Strain	qy17	qy20
	MIC (μg/mL)	MIC (μg/mL)
**Gram-positive bacteria**		
*S. haemolyticus*	16	16
*S. epidermidis*	16	16
*E. enterococcus*	8	16
**Gram-negative bacteria**		
*K. Pneumoniae*	–	–

### Qy17 Inhibits the Biofilm Formation of *Staphylococcus haemolyticus*

Biofilms play pivotal roles in bacterial drug resistance. Here, the inhibitory effect of qy17 and qy20 on the formation of *S. haemolyticus* biofilms was analyzed by crystal violet staining and absorbance value detection. Crystal violet staining showed that both qy17 and qy20 significantly inhibited the biofilm formation of *S. haemolyticus*, and this effect became increasingly obvious as the concentration increased (Figures 3A,D). The absorbance value showed that as the concentrations of qy17 and qy20 increased, the biofilm masses decreased (Figures 3B,E). In addition, gradually increasing the magnification with a Cytation 5, we observed that qy17 and qy20 significantly reduced the biomass and density of the biofilm (Figures 3C,F).

### Qy17 Disrupts Mature *Staphylococcus haemolyticus* Biofilms

It has been suggested that the sensitivity of pathogens to antibacterial agents may be reduced by 10–1,000-fold after the formation of biofilms. Thus, an assay to test the destruction of mature biofilms by qy17 and qy20 was conducted, revealing their ability to disrupt preformed *S. haemolyticus* biofilms. As described above, the crystal violet staining (Figures 4A,D), absorbance (Figures 4B,E) and Cytation 5 (Figures 4C,F) results showed that qy17 and qy20 can both destroy mature *S. haemolyticus* biofilms. This effect became increasingly obvious as the concentration increased.

### Scanning Electron Microscopy Analysis of the *Staphylococcus haemolyticus* Biofilm Structure and Morphology

**FIGURE 10:**
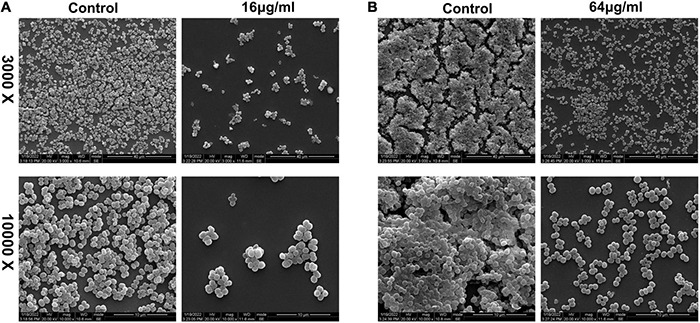
Scanning electron microscopy observations of the effect of qy17 on *S. haemolyticus* biofilms. **(A)** *S. haemolyticus* was incubated with 16 μg/mL qy17 for 24 h. Then, cells on cover slides were collected for SEM observation. **(B)** A 24-h mature biofilm of *S. haemolyticus* was preformed. Then, the mature biofilm was treated with qy17 at a concentration of 64 μg/mL for 24 h. Scanning electron micrographs were taken at 3.00 K and 10.00 K magnifications.

Biofilm formation is a dynamic process. Thus, biofilms can be divided into immature and mature statuses. Here, immature biofilms refer to those grown for less than 24 h to allow the bacteria to form a biofilm with or without qy17 present. To further characterize the role of qy17 in *S. haemolyticus* biofilms, SEM was utilized to analyze the biofilm structure and morphology. The results showed that compared with that in the control group, bacterial aggregation was reduced with qy17 treatment (Figure 10A). Mature biofilm inhibition refers to bacteria grown for 24 h, forming intact biofilms prior to qy17 addition. Notably, qy17 treatment reduced cell aggregation and extracellular aggregate formation, producing a monolayer of adherent cells in mature biofilms. The control group showed *S. haemolyticus* encased in biofilms with multiple layers of adherent cells (Figure 10B).

### Qy17 Affects the Biofilm Formation-Related Signaling Pathways of *Staphylococcus haemolyticus*

To better understand the mechanism of qy17-mediated inhibition of *S. haemolyticus* biofilm formation, the key signaling pathways of biofilm formation were studied. After qy17 treatment, the expression of the *agrB* gene was upregulated, and the expression of the *ebp*, *fbp*, *srtA* and *sigB* genes was downregulated (Figure 5). The detailed results are listed in Supplementary Table 3. Therefore, qy17 inhibited biofilm formation by changing the gene expression of biofilm formation-related signaling pathways in *S. haemolyticus*.

### Qy17 Reduces the Survival of *Staphylococcus haemolyticus* Inside Biofilms

Bacteria encased in the biofilm are potential seeds for new biofilm formation. To further understand the antibiofilm effect of qy17, the MTT and CFU calculation methods were used to determine the number of viable bacteria in qy17-treated *S. haemolyticus* biofilms. After qy17 treatment, the absorbance values and CFUs inside the biofilm gradually decreased in a dose-dependent manner (Figure 6A). After treatment with a high concentration of qy17, the same results were obtained with mature biofilms (Figure 6B). Therefore, with qy17 treatment, the number of viable bacteria inside *S. haemolyticus* biofilms was obviously reduced.

### Transcriptomic Analysis of Differentially Expressed Genes During qy17 Inhibition of *Staphylococcus haemolyticus*

**FIGURE 11:**
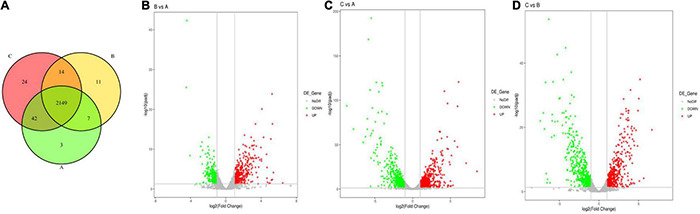
Venn diagram of differential gene expression between the different groups and a volcano plot of the differentially expressed genes (DEGs). *S. haemolyticus* was treated with different concentrations of qy17 for 18 h, and RNA was collected and sequenced. **(A)** Venn diagram of differential gene expression among the different groups (**A**: DMSO, **B**: 8 μg/mL qy17, **C**: 16 μg/mL qy17). **(B–D)** Volcano plot of the significantly differentially expressed genes. (**B**: 8 μg/mL vs. DMSO, **C**: 16 μg/mL vs. DMSO, **D**: 16 μg/mL vs. 8 μg/mL). The y-axis is the negative log10 of the *P*-values (a higher value indicates greater significance), and the x-axis is the log2 fold change or the difference in abundance between the two populations (positive values represent the upregulated genes in the biofilms, and negative values represent downregulated genes).

To investigate the antibacterial mechanism, RNA sequencing was carried out on qy17-treated or untreated *S. haemolyticus* samples. DEG analyses included Venn diagram, volcano diagram, Gene Ontology (GO) and Kyoto Encyclopedia of Genes and Genomes (KEGG) signaling pathway enrichment analyses. Transcriptome analysis showed that many genes in *S. haemolyticus* were differentially regulated in response to qy17 compared with those in the control condition. The Venn diagram showed that the total number of differentially expressed genes (DEGs) in each group (DMSO, 8 μg/mL qy17, and 16 μg/mL qy17) was 2,201, 2,181, and 2,229, respectively (Figure 11A). The volcano chart showed the overall distribution of the DEGs (> 2-fold); the screening criterion was *P* < 0.05. Compared with the DMSO group, the 8 μg/mL qy17 group had a total of 547 DEGs, of which 327 were upregulated and 220 were downregulated. The 16 μg/mL qy17 group had a total of 993 DEGs, of which 515 were upregulated and 478 were downregulated. Compared with the 8 μg/mL group, the 16 μg/mL group had a total of 994 DEGs, of which 486 were upregulated and 508 were downregulated (Figures 11B–D).

**FIGURE 12:**
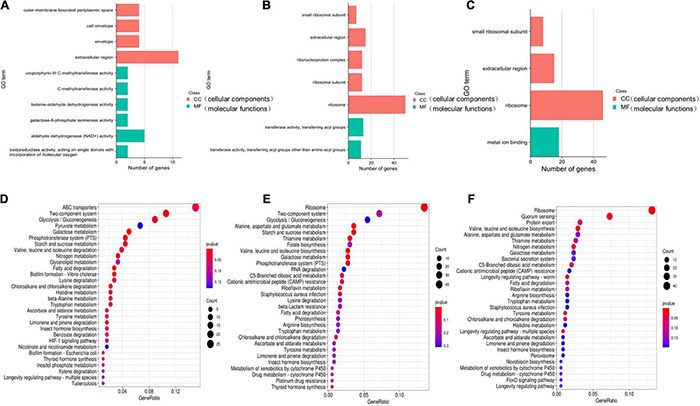
GO and KEGG enrichment analyses of significantly differentially expressed genes. The GO **(A–C)** function-annotated gene distribution map of significantly differentially expressed genes. The differentially expressed genes were divided mainly into two categories: molecular functions (MFs) and cellular components (CCs). KEGG pathway **(D–F)** enrichment bubble chart of significantly differentially expressed genes. GeneRatio refers to the ratio of the number of differentially expressed genes enriched in the pathway (sample number) to the number of annotated genes (background number). The greater the GeneRatio is, the greater the degree of enrichment. The *Q*-value is the *P*-value after multiple hypothesis testing and correction. The range of the *Q*-value is [0,1]. The closer the *Q*-value is to zero, the more significant the enrichment. **(A,D)** 8 μg/mL vs. DMSO. **(B,E)** 16 μg/mL vs. DMSO. **(C,F)** 16 μg/mL vs. 8 μg/mL.

To further understand the functions of these DEGs, GO and KEGG distribution analyses were performed. According to sequence homology, the DEGs were divided into cellular components (CCs) and molecular functions (MFs), and the distribution of DEGs in each GO term was used to explore the differences in CCs and MFs between the experimental group and the control group. The GO classifications showed that the DEGs for the 8 μg/mL group were divided into 38 significantly enriched GO terms, of which 15 terms correspond to MFs and 23 terms correspond to CCs. According to the GO annotations, the main CCs were extracellular region, outer membrane-bounded periplasmic space, cell envelope, and envelopes. The main MF components were aldehyde dehydrogenase (NAD +) activity, uroporphyrin-III C-methyltransferase activity, C-methyltransferase activity, betaine-aldehyde dehydrogenase activity, oxidoreductase activity, action on single donors with the incorporation of molecular oxygen, and galactose-6-phosphate isomerase activity (Figure 12A). The DEGs for the 16 μg/mL group were divided into 120 significantly enriched GO terms, of which 24 terms corresponded to MFs and 96 terms corresponded to CCs. The main CCs were ribosomes, ribosomal subunits, ribonucleoprotein complexes, extracellular regions, and small ribosomal subunits. The main MF components were transferase activity, transfer of acyl groups other than amino-acyl groups, transferase activity, and transfer of acyl groups (Figure 12B). These data indicate that *S. haemolyticus* expresses different genes after exposure to 8 μg/mL and 16 μg/mL qy17 (Figure 12C). Each biological function is accomplished through the cooperation of different genes. The most important biochemical or signaling pathways associated with the DEGs were determined based on the significantly enriched pathways. GO analysis of the DEGs indicated that qy17 affects the cell membrane, redox function and transcription levels of *S. haemolyticus*. KEGG signaling pathway enrichment analysis was performed, and the 30 most abundant pathways were selected. Compared with the control group, the 8 μg/mL group showed altered pathway expression mainly in ABC transporters, two-component systems, glycolysis/gluconeogenesis, and other metabolic pathways. However, the most significantly enriched pathways of the 16 μg/mL group included mainly ribosomes, two-component systems, glycolysis/gluconeogenesis and other metabolic pathways (Figures 12D–F). This finding indicates that qy17 inhibits the energy metabolism pathway required by *S. haemolyticus*, thereby achieving bacteriostatic effects.

**FIGURE 13:**
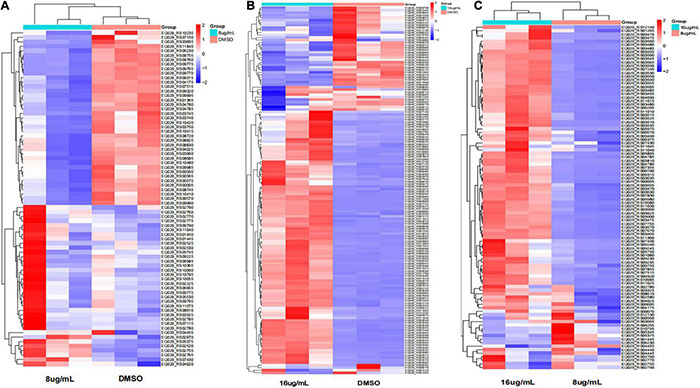
Heatmap of the significantly differentially expressed genes. Red indicates upregulation; blue indicates downregulation; and yellow indicates mildly expressed genes. **(A)** 8 μg/mL vs. DMSO. **(B)** 16 μg/mL vs. DMSO. **(C)** 16 μg/mL vs. 8 μg/mL.

**FIGURE 14:**
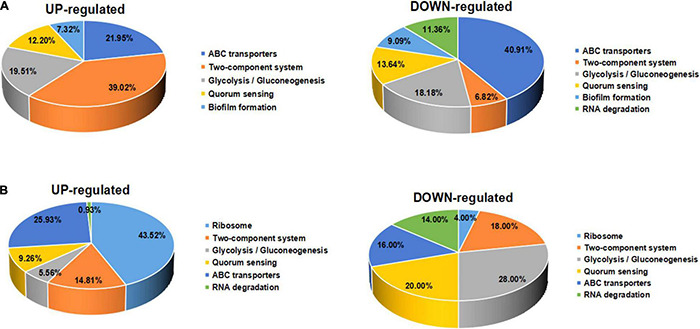
Significantly differentially expressed gene pathway distribution. **(A)** For exposure to qy17 at a concentration of 8 μg/mL, selected upregulated and downregulated genes are displayed in a heatmap. **(B)** For exposure to the compound at an inhibitory concentration of 16 μg/mL, selected upregulated and downregulated genes are displayed in a pie chart.

Furthermore, the expression of genes related to ABC transporters, two-component systems, ribosomes, glycolysis/gluconeogenesis, quorum sensing, biofilm formation, and RNA degradation was analyzed at different drug concentrations using heatmaps (Figures 13A–C). The ID, name and description of the selected genes are shown in Supplementary Table 4. Pie chart analyses showed the distribution of the main genes that were up- or downregulated. After the 8 μg/mL treatment, the upregulated genes were mainly distributed in two-component systems. Downregulated genes were mainly distributed among ABC transporters (Figure 14A). After the 16 μg/mL treatment, the upregulated genes were mainly distributed in the ribosome. Downregulated genes were mainly distributed in glycolysis/gluconeogenesis (Figure 14B). In fact, some biofilm formation-related genes were upregulated in the 8 μg/mL qy17 treatment group (7.32%). However, this effect was not observed in the 16 μg/mL qy17 treatment group (Figure 14). According to reports, low concentrations of antibiotics promote bacterial growth in response to stimuli (Liu et al., 2020). Moreover, we found that a large number of ABC transporter (21.95%) two-component system (39.02%) and glycolysis/gluconeogenesis (19.51%) genes were also upregulated. This result suggests that *S. haemolyticus* selectively expressed these genes, such as ABC transporters, to excrete qy17 to reduce the intracellular drug concentration. In addition, the selective expression of two-component system genes to transmit signals and regulate many downstream genes helps the host to resist environmental stress (Jiang et al., 2019).

### Qy17 Affects Quorum Sensing, Efflux Pumps, Stress Response Systems and Biosynthetic Pathways of *Staphylococcus haemolyticus*

To further validate the transcriptome data, differential gene expression was also verified with RT–PCR. Based on the transcriptomic analysis results, quorum sensing (*yidC*) (Figure 7A), RNA degradation (*rho* and *dnaK*) (Figures 7B,C), ABC transporter (*rdsD*, *rdsC*, and *modA*) (Figures 7D–F), and glycolysis/gluconeogenesis (*gpmI*, *tpiA*, and *gap*) genes were chosen for RT–PCR analysis (Figures 7G–I). The DEG verification results were consistent with the results of the RNA-seq analysis, indicating that RNA-seq successfully identified DEGs. In addition, we also confirmed the expression of *S. haemolyticus* virulence-, stress response- and biotin synthesis-related genes (*clpP*, *clpX*, *clpB*, *groES*, *groL*, *grpE*, *danJ*, *tpiA*, *ahpC*, and *bioB*) after treatment with 16 μg/mL qy17. Their expression levels were significantly reduced (Figures 8A–I).

### Qy17 Affects the Phenol-Soluble Modulin Toxin Gene Expression of *Staphylococcus haemolyticus*

PSM toxins are important virulence factors produced by *S. haemolyticus* that promote sepsis. At the transcriptional level, PSMβ toxin gene expression was downregulated with 16 μg/mL qy17 treatment but upregulated after 8 μg/mL qy17 treatment (Figure 9A). According to the nucleotide sequence of *S. haemolyticus* PSMβ toxin determined in this study, the translation product is the PSMβ toxin peptide that has been previously identified (Table 2). This result may have occurred because a sub-MIC is not enough to kill the bacteria, which can stimulate bacterial rebound growth.

**TABLE 2:** PSMβ-related information.

Gene ID	Description	Nucleotide sequence	Amino acid sequence (N′-C′)	Product
EQ029_RS08170	Phenol-soluble modulin PSM-beta-1	ATGCAAAAATTAGCAGAAGCAATTGCAGCAGCAGT ACAAGCAGGACAAGATAAAGACTGGGGTAAAATGG GTACAAGCATCGTAGGTATCGTAGAAAACGGAA TCAGTGTTTTAGGTAAAATTTTCGGCTTCTAA	MQKLAEAIAAAVQAGQDKDWGKMGT SIVGIVENGISVLGKIFGF	PSMβ1
EQ029_RS08175	Phenol-soluble modulin PSM-beta-2	ATGGAAAAAATCGCAAACGCAGTTAAAAGTGCAATTG AAGCAGGTCAAAACCAAGACTGGACTAAATTAGG TACAAGTATCTTAGATATCGTTTCAAACGGTGTA ACTGAATTAAGTAAAATCTTTGGTTTCTAA	MEKIANAVKSAIEAGQNQDWTKLG TSILDIVSNGVTELSKIFGF	PSMβ2
EQ029_RS08190	Phenol-soluble modulin PSM-beta-3	ATGTCAAAATTAGTACAAGCAATTTCAGATGCAGTT CAAGCAGGCCAAAACCAAGATTGGGCTAAATTAGGTA CAAGCATTGTAGGTATCGTAGAAAACGGTGTT GGCATTTTAGGTAAATTATTCGGATTCTAA	MSKLVQAISDAVQAGQNQDWAKLGT SIVGIVENGVGILGKLFGF	PSMβ3
				

### Qy17 Inhibits the Hemolytic Activity of *Staphylococcus haemolyticus*

After RBCs were treated with different concentrations of qy17, the supernatant color of the drug group was similar to that of the RBC control group, with a lighter RPMI-1640 background color. This result indicates that qy17 does not cause hemolysis of RBCs in the concentration range of 64 μg/mL (Figures 9B,C). *S. haemolyticus* can cause RBC hemolysis and cell death. The protective effect of different concentrations of qy17 on erythrocyte lysis was studied by a hemolysis test. The results showed that the supernatants of the untreated *S. haemolyticus* group and the DMSO-treated group were dark red, and a large number of RBCs were lysed. qy17 could have a certain protective effect, and the color of the supernatant was lighter red than that of the untreated group. In addition, the protective effect increased with increasing drug concentration (Figures 9D,E).

## Discussion

CoNSs are pivotal nosocomial bacteria and one of the main causes of sepsis (Cheung and Otto, 2010; Barros et al., 2012; Jiang et al., 2019; Becker et al., 2020). Among CoNSs, the frequency of *S. haemolyticus* isolation from human blood cultures is slightly lower than that of the most commonly isolated organism, *S. epidermidis* (Falcone et al., 2006; Barros et al., 2012; Farina et al., 2013). *S. haemolyticus* continues to play an important role in hospital-acquired opportunistic infections related to implanted medical devices (Barros et al., 2012; Grant and Hung, 2013; Jiang et al., 2019; Becker et al., 2020). The ability to form biofilms is considered the most important virulence factor for foreign substance-related CoNS infections (Mack et al., 2006; Flemming et al., 2016; Becker et al., 2020). Due to rapidly evolving resistance mechanisms, the treatment of *S. haemolyticus*-related infections is challenging. Natural products are still the main source of drugs for the treatment of bacterial infections (Czekaj et al., 2015; Ciofu and Tolker-Nielsen, 2019; Yong et al., 2019; Mishra et al., 2020). However, it is necessary to understand their mode of action and their molecular interactions with corresponding cellular targets in order to use them as effective tools. Here, we described a promising compound, FA analog qy17, which inhibits *S. haemolyticus* biofilm formation and disrupts mature biofilms. The gene network underlying the mechanism of qy17 antibacterial action was also revealed. The antibacterial effects of qy17 and qy20 against *S. haemolyticus* were preliminarily evaluated based on their MICs (Table 1). The growth curves showed that qy17 and qy20 inhibit the growth of *S. haemolyticus* in a concentration-dependent manner (Figure 2). These results show that qy17 and qy20 are potential natural products for inhibiting *S. haemolyticus*.

Biofilms are a network of microbial communities (Flemming et al., 2016). Their characteristics protect cells from adverse environmental conditions. In addition, their structure makes the resident cells more resistant to desiccation, grazing and antibacterial agents than planktonic cells (Jolivet-Gougeon and Bonnaure-Mallet, 2014; Rabin et al., 2015; Flemming et al., 2016). It is generally believed that preventing biofilm adhesion is a way to solve the biofilm problem (Jolivet-Gougeon and Bonnaure-Mallet, 2014; Mishra et al., 2020). In this study, crystal violet, MTT and CFU counting methods were used to evaluate the antibacterial membrane activity of FA derivatives against *S. haemolyticus*. It was found that qy17 reduced the amount of *S. haemolyticus* biofilm formation (Figure 3) and had a certain disruptive effect on preformed biofilms (Figure 4). SEM further confirmed this result (Figure 10).

EPSs are the main components of bacterial biofilms (Jolivet-Gougeon and Bonnaure-Mallet, 2014; Flemming et al., 2016). Among them, the key matrix components DNA, protein and extracellular polysaccharides are essential to maintain the structural integrity of biofilms and provide shelter for cells (Stewart and Franklin, 2008; Da et al., 2017; Schilcher and Horswill, 2020). In addition to accumulation-associated protein (Aap) and polysaccharide intercellular adhesin (PIA), which are important for biofilm formation, other components, including surface proteins such as fibrinogen-binding protein (*fbp*), elastin-binding protein (*ebp*) and collagen-binding protein (*cbp*), are also considered important factors in biofilms (O’Neill et al., 2008). These cell-surface proteins contain a C-terminal LPXTG motif that can be recognized by sortase A (*srtA*), which helps them adhere to the cell wall (Suree et al., 2009). Inactivation of *srtA* leads to the reduction and cessation of the production of the biofilm matrix (Cascioferro et al., 2015). SrtA plays an important role in cell adhesion, signal transduction and biofilm formation and is considered a universal target for the treatment of gram-positive bacteria (Weiner et al., 2010).

The absence of the quorum sensing system regulator *agr* can enhance the formation of *S. aureus* biofilms (Vuong et al., 2000; Coelho et al., 2008; Xu et al., 2017; Paulander et al., 2018). The transcription factor *sigB* can upregulate the factors necessary for the early stage of biofilm formation, including aggregation factors and fibronectin-binding protein (Nair et al., 2003). Protein, DNA or both are the main components of most *S. haemolyticus* biofilm matrices (Panda and Singh, 2018). In this study, after qy17 treatment, the expression of surface proteins of *S. haemolyticus* biofilms, such as fibrinogen-binding protein (*fbp*), elastin-binding protein (*ebp*), global regulatory factor (*sigB*), and sortase A (*srtA*), was attenuated. The quorum signal sensing system (*agrB*) was upregulated (Figure 5). It can be inferred that qy17’s inhibition of *S. haemolyticus* biofilm formation may occur through the downregulation of transcription factors (*sigB*), the sortase gene (*srtA*), and bacterial surface proteins (*fbp*, *ebp*) and the upregulation of density-sensing system genes (*agrB*). To further verify the antibacterial function of qy17, the viability of *S. haemolyticus* inside the biofilm was detected. Intriguingly, qy17 also attenuated the survival of bacteria encased in the biofilm and reduced the number of intrabiofilm *S. haemolyticus* (Figure 6). Although the disruption of the mature biofilm resulted in an obvious difference in only the OD value (Figure 4B), the bacterial CFUs inside the mature biofilm were significantly decreased upon qy17 treatment (Figure 6B). These results suggest that qy17 may penetrate into the mature biofilm and inhibit *S. haemolyticus.* Thus, it is reasonable to anticipate that qy17 may reduce the ability of biofilm-resident bacteria to seed new biofilm formation elsewhere when the biofilm matures.

After the biofilm is fully mature, the bacterial cells residing inside it release certain chemicals to break and disperse the biofilm (Jolivet-Gougeon and Bonnaure-Mallet, 2014; Flemming et al., 2016). These planktonic cells are ready to either recolonize the same site or attach to a different site and repeat the process to form a new biofilm (Grant and Hung, 2013; Jolivet-Gougeon and Bonnaure-Mallet, 2014; Flemming et al., 2016). Bacterial viability decreased in qy17-treated *S. haemolyticus* biofilms (Figure 6). This result shows that the bacteria encased by the biofilm that would seed new biofilm formation are destroyed and that their colonization of new sites is inhibited, which inhibits new biofilm formation.

To further study how the compounds inhibit *S. haemolyticus*, qy17, which had a better antibacterial effect, was chosen for transcriptome sequencing and analysis and for subsequent experiments. Differential gene expression data indicated that qy17 mainly exerts antibacterial effects by regulating ABC transporters, ribosomes, two-component systems, glycolysis/gluconeogenesis and other pathways of *S. haemolyticus*. Qy17 induced or inhibited the transcription of genes encoding proteins and enzymes involved in metabolic pathways (Figures 11, 12). The upregulated genes were mainly involved in key metabolic pathways required for bacterial survival, while the downregulated genes belonged to bacterial transport systems and bacterial adhesion (Figures 13, 14). qRT–PCR results further verified the transcriptome findings, and quorum sensing (*yidC*), RNA degradation (*rho*, *dnaK*), ABC transporter (*rdsD*, *rdsC*, *modA*), and glycolysis/gluconeogenesis (*gpmI*, *tpiA*, *gap*) pathway-related gene expression levels were altered. This result shows that the transcriptome results are reliable (Figure 7).

Among the DEG-encoded proteins, the ATP-binding cassette (ABC) transporter is an efflux pump that participates in the influx or efflux of a large number of molecules (Orelle et al., 2019). It uses the energy of ATP hydrolysis to expel drugs and antibiotics and is a multidrug efflux pump. Multidrug efflux transporters are a major problem in antibiotic resistance, as they provide bacteria with the ability to evade most current therapies. Qy17 treatment downregulated ABC transporters (rdsD, rdsC, modA) (Figures 7D–F). Therefore, the antibacterial effects of qy17 and its inhibition of the bacterial efflux process may have the potential to treat infections caused by drug-resistant *S. haemolyticus* (Panda and Singh, 2018).

The physiological tolerance of biofilms for various antibacterial agents is generally due to the induction of adaptive SOS responses by persistent bacteria deep in the biofilm, thereby making the bacteria more tolerant (Grant and Hung, 2013; Jolivet-Gougeon and Bonnaure-Mallet, 2014; Ciofu and Tolker-Nielsen, 2019; Podlesek and Zgur Bertok, 2020). It is well known that the persisting bacteria inside mature biofilms have lower metabolic activity (Grant and Hung, 2013). Nevertheless, we tested the transcriptome changes in nascent biofilms (less than 24 h) and all the bacteria in this study. Because biofilm formation is a dynamic process, bacterial migration and biofilm release occur along with biofilm formation. Planktonic bacteria can also form new biofilms under proper conditions. Thus, all bacterial biofilm cells (biofilm-resident bacteria plus planktonic bacteria) were collected for RNA isolation to measure the biofilm formation process. Upon qy17 challenge, many stresses response genes were downregulated (total expression level). After 16 μg/mL qy17 treatment, the expression of regulatory Clp protease-related genes (*clpP*, *clpX*, and *clpB*) and stress response genes (*groES*, *groL*, *grpE*, *dnaK*, dnaJ, and *aphC*) in *S. haemolyticus* was significantly downregulated (Figure 8). According to reports, when bacteria enter the host, they are immediately exposed to different environments, including changes in temperature, osmotic pressure, and pH. Bacteria adhere, invade or escape these stimuli by increasing the expression of virulence factors and stress response proteins, including heat shock proteins and chaperone proteins (Fourie and Wilson, 2020). Bacteria usually use these chaperones and proteases to facilitate a compensatory response to stress conditions in order to survive (Frees et al., 2014). The Clp ATPase enzyme and Clp protease play a central role in the stress response required to manage adverse host conditions. Bacterial ClpB is an ATP-dependent aggregating enzyme. In recent decades, many studies have focused on the activation of the dual-chaperone system of stress-aggregating proteins. This system is composed mainly of the chaperone systems DnaK/DnaJ/GrpE and ClpB (Zolkiewski, 1999). Under stress conditions, the Clp protein and its companion proteins, such as DnaK, GroEL, ClpB, and ClpC, can refold denatured proteins (Zolkiewski, 1999; Frees et al., 2014; Zolkiewski et al., 2016; Fourie and Wilson, 2020). ClpB is necessary for bacteria to survive under environmental stress (Zolkiewski, 1999). It cooperates with the DnaK chaperone system to reactivate aggregated proteins, allowing bacteria to survive under environmental conditions such as high temperature and oxidative stress (Grant and Hung, 2013; Fourie and Wilson, 2020; Mishra et al., 2020; Podlesek and Zgur Bertok, 2020). BioB is a biotin synthetase, and biotin plays a pivotal role in bacterial survival *via* cell metabolism, such as carboxylation, decarboxylation and transcarboxylation reactions (Satiaputra et al., 2016). After qy17 treatment, these genes (*clpB*, *groES*, *groL*, *grpE*, *dnaK*, dnaJ, *aphC*, and *bioB*) in *S. haemolyticus* were downregulated. This result indicates that qy17 inhibits stress response-related genes, causing *S. haemolyticus* to be unable to compensate for various stress conditions, which in turn affects the growth and survival of the bacteria.

Furthermore, the Clp protease can regulate bacterial virulence and degrade the antitoxin in toxin–antitoxin systems (TASs) (Ju et al., 2021). TASs play an important role in the formation of biofilms and persistence (Jolivet-Gougeon and Bonnaure-Mallet, 2014; Ciofu and Tolker-Nielsen, 2019; Mishra et al., 2020). In *S. aureus*, ClpCP promotes the decomposition of antitoxins. The absence of *clpP*, *clpC* and *clpX* in *S. aureus* leads to a decrease in persistence. Targeting the ClpP protease can interfere with the biological process of TASs, which can eliminate chronic biofilm infections and inhibit persistent biofilms (Frees et al., 2003; Jenul and Horswill, 2019; Schelin et al., 2020; Ju et al., 2021). The contribution of the Clp protein to toxicity may occur at multiple levels (Kedzierska et al., 2003). *S. aureus* has a peptidase subunit (ClpP) and an ATP enzyme chaperone (ClpX or ClpC) (Ye et al., 2016). ClpP and ClpX control the expression of major virulence factors and the transcription of protein A in *S. aureus* (Mei et al., 1997; Ye et al., 2016). In clpX or clpP mutants, the transcription levels of the virulence genes *hla, sspA*, and *spa* are reduced (Mei et al., 1997; Frees et al., 2014; Jenul and Horswill, 2019). In addition, in a mouse skin abscess model, the inactivation of *clpX* and *clpP* completely reduced the formation of abscesses (Jenul and Horswill, 2019).

PSMs are a family of staphylococcal peptide toxins that promote inflammation and cell lysis (Xu et al., 2017). PSMs have a significant contribution to disease manifestations caused by *S. aureus* (Cheung et al., 2014). Recently, three PSMβ peptides (PSMβ1, PSMβ2, and PSMβ3) with hemolytic ability were detected in *S. haemolyticus* (Da et al., 2017). Interestingly, 8 μg/mL qy17 treatment upregulated the expression of these genes. This phenomenon may have occurred because treatment with a low concentration or a sub-MIC of a drug (antibiotic stress), bacteria attempt to express virulence genes for survival, as described above, as well as stress response genes (heat and oxidative stress). These results are consistent with Dr. Shen and Zhu’s previous report “Sublethal Levels of Antibiotics Promote Bacterial Persistence in Epithelial Cells” (Liu et al., 2020). However, after 16 μg/mL qy17 treatment, the regulation of PSMβ-related genes in *S. haemolyticus* was downregulated (Figure 9). Moreover, the amino acid sequence obtained from these PSMβ gene sequences is consistent with the identified PSMβ toxin (Da et al., 2017). Qy17 inhibits the expression of the PSMβ gene at the transcriptional level, resulting in the reduction of the levels of PSMβ1, PSMβ2, and PSMβ3 toxins (Table 2). Qy17 inhibited the hemolytic activity of *S. haemolyticus* (Figures 9B–E). This finding further indicates that the MIC of qy17 may inhibit the secretion of PSM toxins by *S. haemolyticus*, leading to weakened pathogenicity. This effect may have a significant impact on the treatment of blood infections and sepsis caused by *S. haemolyticus*.

**FIGURE 15:**
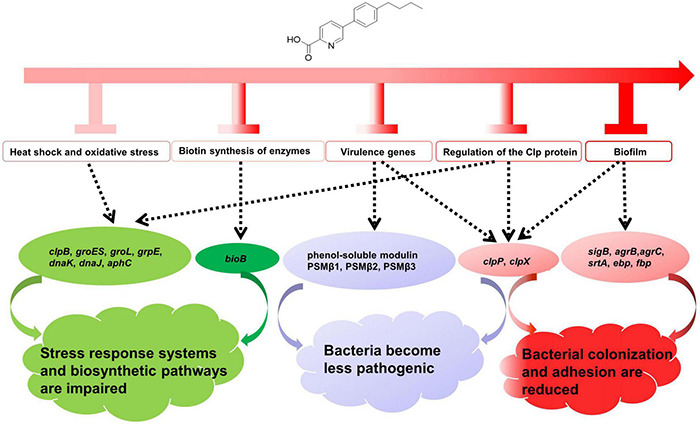
Overview of the antibacterial mechanisms of qy17 against *S. haemolyticus.* (i) Regulation of genes related to the stress response, heat shock and biotin response causes *S. haemolyticus* to be unable to compensate for various stress conditions, thereby affecting the growth and survival of the bacteria. (ii) Regulation of the expression of virulence genes influences bacterial pathogenicity. (iii) Regulation of biofilm-related genes affects bacterial colonization, adhesion and biofilm formation.

The antibacterial mechanism of qy17 against *S. haemolyticus* is mediated by several pathways, as shown in Figure 15. These include (i) the inhibition of genes related to the stress response, heat shock and biotin response, which resulted in *S. haemolyticus* being unable to compensate for various stress conditions, thereby affecting the growth and survival of the bacteria; (ii) reduced expression of virulence genes that affect the pathogenicity of the bacteria; (iii) the inhibition and destruction of biofilms, regulation of the expression of biofilm-related genes, and an effect on bacterial colonization and adhesion; and (iv) reduced number and viability of bacteria inside the biofilm and reduction in the number and source of seeds that form new biofilms (Figure 15). In summary, we believe that qy17 inhibits *S. haemolyticus* and disrupts its biofilm formation. This study provides new drug candidates and a theoretical basis for the clinical treatment of infections caused by *S. haemolyticus*.

## Data Availability Statement

The original contributions presented in the study are publicly available. This data can be found here: https://www.ncbi.nlm.nih.gov/bioproject, PRJNA799664.

## Author Contributions

BW designed and implemented the experiments of this study and drafted the manuscript. C-RS performed the experiments, analyzed the data, and prepared the article. Q-YZ and G-BX synthesized the compounds. P-WW and XW performed the experiments and analyzed the data. S-GL guided the synthesis experiment. Y-HL, Y-XY, and H-ML guided the RNA and transcriptomic experiments and data analysis. G-BX and H-ML analyzed the data and assisted with the design. All authors assisted in editing the manuscript.

## Conflict of Interest

The authors declare that the research was conducted in the absence of any commercial or financial relationships that could be construed as a potential conflict of interest.

## Publisher’s Note

All claims expressed in this article are solely those of the authors and do not necessarily represent those of their affiliated organizations, or those of the publisher, the editors and the reviewers. Any product that may be evaluated in this article, or claim that may be made by its manufacturer, is not guaranteed or endorsed by the publisher.

## Supplementary Material

Supplementary Table 1 *S. haemolyticus* transcriptome-related gene sequence primer list.

Supplementary Table 2 *S. haemolyticus* biofilm gene sequence primer list.

Supplementary Table 3 Biofilm-related gene list.

Supplementary Table 4 Stress response gene list.

Supplementary Table 5 Stress response gene list.
