# Supplementary material for: The Fusaric Acid Derivative qy17 Inhibits Staphylococcus haemolyticus by Disrupting Biofilm Formation and the Stress Response via Altered Gene Expression
Source: Front Microbiol. 2022 Mar 14;13:822148. doi: 10.3389/fmicb.2022.822148 (PMC8964301; doi:10.3389/fmicb.2022.822148)
Supplement: Supplementary Table 1 — S. haemolyticus transcriptome-related gene sequence primer list. [file Data_Sheet_1.docx]

**The IR, NMR and HR-MS data of fusaric acid derivatives**

**qy17：*5-(4-Butylphenyl) picolinic acid***: White solid; IR (KBr): υ 3441, 3076, 2926, 1694, 1590, 1389, 1282, 1226, 1153, 1001, 798 cm^-1^. ^1^H NMR (600 MHz, CDCl_3_) δ: 8.94 (s, 1H), 8.27 (d, *J* = 7.7 Hz, 1H), 8.10 (d, *J* = 7.7 Hz, 1H), 7.55 (d, *J* = 8.1 Hz, 2H), 7.35 (d, *J* = 8.1 Hz, 2H), 2.66 (t, *J* = 7.7 Hz, 2H), 1.62 (m, 2H), 1.36 (m, 2H), 0.93 (t, d, *J* = 7.7 Hz, 3H). ^13^C NMR (125 MHz, CDCl_3_) δ: 164.8, 146.7, 144.8, 144.7, 141.1, 136.4, 133.6, 129.7 (C×2), 127.4 (C×2), 124.4, 35.6, 33.7, 22.5, 14.1. HRMS-ESI: *m/z* 256.1333 [M+H]^+^ (Calcd for C_16_H_18_NO_2_, 256.1332).

**qy20：*5-(4-(tert-Butyl) phenyl)picolinic acid***: White solid; IR (KBr): υ 3430, 3031, 2961, 1699, 1629, 1588, 1387, 1289, 1228, 1150, 1114, 1002, 836 cm^-1^. ^1^H NMR (600 MHz, CDCl_3_) δ: 8.94 (s, 1H), 8.27 (d, *J* = 7.8 Hz, 1H), 8.10 (d, *J* = 7.8 Hz, 1H), 7.58 (m, 2H), 7.53 (m, 2H), 1.35 (s, 9H). ^13^C NMR (125 MHz, CDCl_3_) δ: 164.7, 152.7, 146.5, 144.5, 140.8, 136.2, 133.2, 127.1 (C×2), 126.4 (C×2), 124.1, 34.8, 31.3 (C×3). HRMS-ESI: *m/z* 256.1333 [M+H]^+^ (Calcd for C_16_H_18_NO_2_, 256.1332).

**Supplementary Table 1** *Staphylococcus haemolyticus* biofilm gene sequence primer list.

| Gene | Sequence (5' to 3') | bp |
| --- | --- | --- |
| *sigB* F | CCGACCGTTATTGGTGAGAT | 20 |
| *sigB* R | ACGGTCAGCAATTTCTGC | 18 |
| *agrB* F | GTTGCTGAAGGAGCATAG | 18 |
| *agrB* R | TTATGCACACGGTGCACATGCA | 22 |
| *agrC* F | CGACTAAGGTCAATCGTG | 18 |
| *agrC* R | CGAGAAGATGATATGCCAGG | 20 |
| *srtA* F | CACCAACCATCCCTAAGGAT | 20 |
| *srtA* R | GGTAATGTGGTCTATCGG | 18 |
| *ebp* F | AAGACGCTGCAATAGCTGGA | 20 |
| *ebp* R | ACGATGGTCGTCATGAAGGT | 20 |
| *fbp* F | CGCGTTAAACAACGCGCAAA | 20 |
| *fbp* R | ACAGTTACAGCGTCATCACC | 20 |

**Supplementary Table 2** Transcriptome-related gene sequences primer list of *Staphylococcus haemolyticus*

| Gene | Sequence (5' to 3') | bp |
| --- | --- | --- |
| *yidC* F | GCCAGTTGTCATGGGATTATACTTTG | 26 |
| *yidC* R | GAATGCCGCACTGACTGACC | 20 |
| *rbsD* F | AGACGTTAAGAAGATTGACCTTGC | 24 |
| *rbsD* R | TATGGCGTTGCTTCCCCTG | 19 |
| *rbsC* F | GTAAGGGCTACTTTATCGGTATTC | 24 |
| *rbsC* R | GCAGCAATAGCGTCTAACTCATAAG | 25 |
| *modA* F | CTTAGAAGATCAAGGTTTATACGGCG | 26 |
| *modA* R | GCAAGTTTCTTATCTGAAGTAGCACCTG | 28 |
| *eno* F | GCACCAATCGCATTCCAAG | 19 |
| *eno* R | CAGATGATGCACAGTCAAATCCTAAG | 26 |
| *gpmI* F | GCAGTTATCTTTTTCAACTTCCGC | 24 |
| *gpmI* R | GATTAAACGACGACGCTCACC | 21 |
| *tpiA* F | CAGAACGTCGTGAATTATTCCATG | 24 |
| *tpiA* R | CAACATCTTGACTAGCCACTTTCG | 24 |
| *gap* F | GTTGTTGATGGTGGTTTCCG | 20 |
| *gap* R | GTCATTGTGGTTAGTGTTGAATACG | 25 |
| *rho* F | AGCCTGATGGTTATGGGTTCC | 21 |
| *rho* R | TAGACCAATAGGCGTAACTAAATCC | 25 |
| *dnaK* F | TGCCGAAGAAAACGCTGAAG | 20 |
| *dnaK* R | CGCTTTAATATCTTCGATGTCTTGTCC | 27 |
| *clpP* F | TCTCCAGGTGGTAGTGTCACTGC | 23 |
| *clpP* R | GATTTCAGTTGCTTGTCCTTGTGC | 24 |
| *clpX* F | TAGGTGGTGCGTTTGATGGC | 20 |
| *clpX* R | TTACTAAAGCGTTCTTAGGTTGCG | 24 |
| *clpB* F | AACAAGCGTCTTTACAATCTCGTG | 24 |
| *clpB* R | GAATCATGCGTTCGCTGTCA | 20 |
| *groE* F | AGTAGCTGTTGGAACTGGACGAG | 23 |
| *groE* R | CTCCACGTTTAACTTCTGAGCCAG | 24 |
| *gro*L F | ATCGCTGAAAATGCTGGGCTA | 21 |
| *groL* R | GTTAAGAACATCGCAGCAACACTTG | 25 |
| *grpE* F | TTGATAATATTGAACGCGCACTAC | 24 |
| *grpE* R | TGGATTATCATCTTGAACAACAGCC | 25 |
| *dnaJ* F | AGGAACACCAGGAGAAAACGG | 21 |
| *dnaJ* R | GCCAGAAGGAATTGTCAACATAAC | 24 |
| *bioB* F | CGATATGGCATTTGCGTTGAG | 21 |
| *bioB* R | TTGGCTGCTTTTAATGCGATGG | 22 |
| *ahpC F* | AGGTTCTTGGAGCGTAGTATGTTTC | *25* |
| *ahpC* R | ACGAGTGATTGTTTGAGAAGGGTC | 24 |

**Supplementary Table 3**

| Name | Description | function in biofilms |
| --- | --- | --- |
| *sigB* | RNA polymerase sigma factor SigB | Up-regulation of factors necessary for the early stage of *Staphylococcus aureus* biofilm formation |
| *agrB* | accessory gene regulator AgrB | Up-regulation of dissociation factors, down-regulation of colonization factors |
| *agrC* | accessory regulator AgrC | Up-regulation of dissociation factors, down-regulation of colonization factors |
| *srtA* | class A sortase SrtA | binds extracellular matrix proteins |
| *ebp* | elastin binding protein | Promotes biofilm formation by increasing intercellular aggregation |
| *fbp* | fibrinogen binding protein | Promotes biofilm formation by increasing intercellular aggregation |

**Supplementary Table 4** Transcriptome heatmap gene list.

**A :** 8 μg/mL VS DMSO

| **Gene ID** | Name | log2FC | Description |
| --- | --- | --- | --- |
| **ABC transporters** | | | |
| EQ029_RS00560 | *rbsD* | -1.14204691 | D-ribose pyranase |
| EQ029_RS00565 | *-* | -1.188083132 | sugar ABC transporter ATP-binding protein |
| EQ029_RS00570 | *rbsC* | -1.610668583 | ribose ABC transporter permease |
| EQ029_RS00575 | *-* | -1.191123206 | D-ribose ABC transporter substrate-binding protein |
| EQ029_RS02520 | *-* | 2.831589305 | ABC transporter ATP-binding protein |
| EQ029_RS02525 | *-* | 1.392511221 | ABC transporter permease |
| EQ029_RS02530 | *-* | 1.222334377 | osmoprotectant ABC transporter substrate-binding protein |
| EQ029_RS02970 | *-* | 2.836912256 | FtsX-like permease family protein |
| EQ029_RS03325 | *modA* | 2.270540225 | molybdate ABC transporter substrate-binding protein |
| EQ029_RS03740 | *-* | -1.503548242 | ABC transporter substrate-binding protein |
| EQ029_RS03745 | *-* | -1.376310474 | iron ABC transporter permease |
| EQ029_RS03750 | *-* | -1.490302918 | iron chelate uptake ABC transporter family permease subunit |
| EQ029_RS07115 | *-* | 2.426183105 | PstS family phosphate ABC transporter substrate-binding protein |
| EQ029_RS07150 | *-* | -2.048817558 | ABC transporter substrate-binding protein |
| EQ029_RS08685 | *-* | -1.298146939 | ABC transporter permease |
| EQ029_RS08825 | *-* | -1.236478469 | ABC transporter ATP-binding protein |
| EQ029_RS08830 | *-* | -1.035379092 | energy-coupling factor transporter transmembrane protein EcfT |
| EQ029_RS09225 | *-* | 1.018408037 | peptide ABC transporter substrate-binding protein |
| EQ029_RS09985 | *-* | -1.410212646 | siderophore ABC transporter substrate-binding protein |
| EQ029_RS09990 | *-* | -1.297768326 | ATP-binding cassette domain-containing protein |
| EQ029_RS09995 | *-* | -1.411177035 | iron chelate uptake ABC transporter family permease subunit |
| EQ029_RS10055 | *-* | 1.21888488 | ABC transporter permease/substrate-binding protein |
| EQ029_RS10060 | *-* | 1.548467274 | ABC transporter ATP-binding protein |
| EQ029_RS10235 | *-* | -1.267521298 | ABC transporter ATP-binding protein/permease |
| EQ029_RS10410 | *-* | -1.085597916 | iron ABC transporter permease |
| EQ029_RS10415 | *-* | -1.083559593 | iron ABC transporter permease |
| EQ029_RS10420 | *-* | -1.100892555 | ABC transporter ATP-binding protein |
| **Two-component system** | | | |
| EQ029_RS00005 | *-* | -1.789712844 | chromosomal replication initiator protein DnaA |
| EQ029_RS02755 | *-* | 3.128322539 | nitrate reductase subunit alpha |
| EQ029_RS02760 | *narH* | 2.597539811 | nitrate reductase subunit beta |
| EQ029_RS02765 | *narJ* | 3.150455761 | nitrate reductase molybdenum cofactor assembly chaperone |
| EQ029_RS02770 | *narI* | 2.445336401 | respiratory nitrate reductase subunit gamma |
| EQ029_RS02775 | *nreA* | 2.031739337 | nitrate respiration regulation accessory nitrate sensor NreA |
| EQ029_RS02780 | *nreB* | 1.742050513 | nitrate respiration regulation sensor histidine kinase NreB |
| EQ029_RS02785 | *nreC* | 2.058399109 | nitrate respiration regulation response regulator NreC |
| EQ029_RS02790 | *-* | 1.827252448 | NarK/NasA family nitrate transporter |
| EQ029_RS04455 | *-* | 1.512498537 | accessory gene regulator AgrB |
| EQ029_RS04780 | *-* | -1.377381505 | sensor histidine kinase |
| EQ029_RS04785 | *vraR* | -1.534757888 | two-component system response regulator VraR |
| EQ029_RS05745 | *-* | 1.080718628 | response regulator transcription factor |
| EQ029_RS06985 | *-* | 1.004441063 | response regulator transcription factor |
| EQ029_RS07115 | *-* | 2.426183105 | PstS family phosphate ABC transporter substrate-binding protein |
| EQ029_RS07430 | *-* | 2.703197365 | sensor histidine kinase |
| EQ029_RS08610 | *-* | 1.697901297 | heme A synthase |
| EQ029_RS10365 | *graR* | 1.231477086 | response regulator transcription factor GraR/ApsR |
| EQ029_RS10795 | *-* | 1.448101818 | acetyl-CoA C-acyltransferase |
| Glycolysis / Gluconeogenesis | | | |
| EQ029_RS00775 | *lpdA* | 1.324788834 | dihydrolipoyl dehydrogenase |
| EQ029_RS00790 | *-* | 1.722511499 | 2-oxo acid dehydrogenase subunit E2 |
| EQ029_RS00795 | *lpdA* | 1.308525437 | dihydrolipoyl dehydrogenase |
| EQ029_RS01365 | *-* | -1.574226711 | PTS transporter subunit EIIC |
| EQ029_RS01450 | *-* | 1.465593574 | alcohol dehydrogenase catalytic domain-containing protein |
| EQ029_RS02225 | *-* | 1.331712975 | aldehyde dehydrogenase family protein |
| EQ029_RS04020 | *-* | 1.82526486 | aldehyde dehydrogenase family protein |
| EQ029_RS05530 | *acsA* | 1.32862985 | acetate--CoA ligase |
| EQ029_RS06955 | *-* | 1.384218582 | PTS glucose transporter subunit IIA |
| EQ029_RS08720 | *-* | -1.078547977 | alpha-ketoacid dehydrogenase subunit beta |
| EQ029_RS09755 | *eno* | -1.845639235 | phosphopyruvate hydratase |
| EQ029_RS09760 | *gpmI* | -2.042922228 | 2,3-bisphosphoglycerate-independent phosphoglycerate mutase |
| EQ029_RS09765 | *tpiA* | -1.816818614 | triose-phosphate isomerase |
| EQ029_RS09770 | *-* | -2.114994548 | phosphoglycerate kinase |
| EQ029_RS09775 | *gap* | -1.642819481 | type I glyceraldehyde-3-phosphate dehydrogenase |
| EQ029_RS11840 | *-* | -4.409144178 | aldehyde dehydrogenase family protein |
| **Quorum sensing** | | | |
| EQ029_RS01445 | *-* | 1.477432162 | N-acyl homoserine lactonase family protein |
| EQ029_RS03665 | *lacD* | -2.343320462 | tagatose-bisphosphate aldolase |
| EQ029_RS03960 | *-* | -1.067682678 | S-ribosylhomocysteine lyase |
| EQ029_RS04175 | *yidC* | -1.095372406 | membrane protein insertase YidC |
| EQ029_RS04455 | *-* | 1.512498537 | accessory gene regulator AgrB |
| EQ029_RS05375 | *-* | 2.380920406 | MarR family transcriptional regulator |
| EQ029_RS07515 | *hfq* | -1.160582873 | RNA chaperone Hfq |
| EQ029_RS09225 | *-* | 1.018408037 | peptide ABC transporter substrate-binding protein |
| EQ029_RS09315 | *lepB* | -1.102279689 | signal peptidase I |
| EQ029_RS09320 | *lepB* | -1.533973708 | signal peptidase I |
| EQ029_RS11040 | *secE* | 2.223545806 | preprotein translocase subunit SecE |
| **Biofilm formation** | | | |
| EQ029_RS03960 | *-* | -1.067682678 | S-ribosylhomocysteine lyase |
| EQ029_RS06955 | *-* | 1.384218582 | PTS glucose transporter subunit IIA |
| EQ029_RS07515 | *hfq* | -1.160582873 | RNA chaperone Hfq |
| EQ029_RS10480 | *-* | -1.138469073 | WecB/TagA/CpsF family glycosyltransferase |
| EQ029_RS11075 | *cysE* | 1.165704106 | serine O-acetyltransferase |
| EQ029_RS03960 | *-* | -1.067682678 | S-ribosylhomocysteine lyase |
| EQ029_RS06955 | *-* | 1.384218582 | PTS glucose transporter subunit IIA |
| RNA degradation |  |  |  |
| EQ029_RS04025 | *rho* | -1.021577238 | transcription termination factor Rho |
| EQ029_RS06230 | *dnaK* | -1.497481317 | molecular chaperone DnaK |
| EQ029_RS07515 | *hfq* | -1.160582873 | RNA chaperone Hfq |
| EQ029_RS08745 | *-* | -1.089352031 | ribonuclease J |
| EQ029_RS09755 | *eno* | -1.845639235 | phosphopyruvate hydratase |

**B :** 16 μg/mL VS DMSO

| Gene ID | Name | log2FC | Description |
| --- | --- | --- | --- |
| Ribosome | | | |
| EQ029_RS00070 | *rplI* | -1.849901659 | 50S ribosomal protein L9 |
| EQ029_RS03465 | *rpsJ* | 3.830333984 | 30S ribosomal protein S10 |
| EQ029_RS03470 | *rplC* | 1.947099076 | 50S ribosomal protein L3 |
| EQ029_RS03475 | *rplD* | 2.645647061 | 50S ribosomal protein L4 |
| EQ029_RS03480 | *rplW* | 2.898212601 | 50S ribosomal protein L23 |
| EQ029_RS03485 | *rplB* | 3.270750114 | 50S ribosomal protein L2 |
| EQ029_RS03490 | *rpsS* | 1.322902207 | 30S ribosomal protein S19 |
| EQ029_RS03495 | *rplV* | 3.6424206 | 50S ribosomal protein L22 |
| EQ029_RS03500 | *rpsC* | 2.435215902 | 30S ribosomal protein S3 |
| EQ029_RS03505 | *rplP* | 3.170007896 | 50S ribosomal protein L16 |
| EQ029_RS03510 | *rpmC* | 4.95015878 | 50S ribosomal protein L29 |
| EQ029_RS03515 | *rpsQ* | 2.637918065 | 30S ribosomal protein S17 |
| EQ029_RS03525 | *rplX* | 1.948495572 | 50S ribosomal protein L24 |
| EQ029_RS03530 | *rplE* | 2.91606128 | 50S ribosomal protein L5 |
| EQ029_RS03535 | *-* | 4.872781135 | type Z 30S ribosomal protein S14 |
| EQ029_RS03540 | *rpsH* | 2.91832689 | 30S ribosomal protein S8 |
| EQ029_RS03545 | *rplF* | 3.027712934 | 50S ribosomal protein L6 |
| EQ029_RS03550 | *rplR* | 3.92694528 | 50S ribosomal protein L18 |
| EQ029_RS03555 | *rpsE* | 2.238617888 | 30S ribosomal protein S5 |
| EQ029_RS03560 | *rpmD* | 1.679809689 | 50S ribosomal protein L30 |
| EQ029_RS03565 | *rplO* | 2.071812409 | 50S ribosomal protein L15 |
| EQ029_RS03585 | *rpmJ* | 2.005509449 | 50S ribosomal protein L36 |
| EQ029_RS03590 | *rpsM* | 1.680339582 | 30S ribosomal protein S13 |
| EQ029_RS03595 | *rpsK* | 2.673994263 | 30S ribosomal protein S11 |
| EQ029_RS03630 | *rplM* | 1.042710789 | 50S ribosomal protein L13 |
| EQ029_RS03635 | *rpsI* | 2.439084437 | 30S ribosomal protein S9 |
| EQ029_RS04030 | *-* | 3.152916706 | type B 50S ribosomal protein L31 |
| EQ029_RS05600 | *rpsD* | 2.829611664 | 30S ribosomal protein S4 |
| EQ029_RS05805 | *rpmI* | 3.448098345 | 50S ribosomal protein L35 |
| EQ029_RS05810 | *rplT* | 3.718522175 | 50S ribosomal protein L20 |
| EQ029_RS06205 | *rpsT* | 1.608812377 | 30S ribosomal protein S20 |
| EQ029_RS06255 | *rpsU* | -1.317312566 | 30S ribosomal protein S21 |
| EQ029_RS06375 | *rpmG* | 2.778363905 | 50S ribosomal protein L33 |
| EQ029_RS07355 | *rpmG* | 2.665020856 | 50S ribosomal protein L33 |
| EQ029_RS07670 | *rpsO* | 1.599334755 | 30S ribosomal protein S15 |
| EQ029_RS07755 | *rpsB* | 1.919441216 | 30S ribosomal protein S2 |
| EQ029_RS07830 | *rplS* | 1.730017117 | 50S ribosomal protein L19 |
| EQ029_RS07845 | *rpsP* | 2.977680496 | 30S ribosomal protein S16 |
| EQ029_RS07920 | *rpmB* | 1.177492345 | 50S ribosomal protein L28 |
| EQ029_RS10980 | *rpsG* | 1.853543615 | 30S ribosomal protein S7 |
| EQ029_RS10985 | *rpsL* | 2.324015639 | 30S ribosomal protein S12 |
| EQ029_RS11010 | *rplL* | 4.57410318 | 50S ribosomal protein L7/L12 |
| EQ029_RS11015 | *-* | 4.620004623 | 50S ribosomal protein L10 |
| EQ029_RS11025 | *rplA* | 2.663932013 | 50S ribosomal protein L1 |
| EQ029_RS11030 | *rplK* | 2.0750178 | 50S ribosomal protein L11 |
| EQ029_RS11300 | *-* | 2.19547808 | 50S ribosomal protein L25/general stress protein Ctc |
| EQ029_RS11955 | *rpsR* | 1.871094564 | 30S ribosomal protein S18 |
| EQ029_RS11965 | *-* | 1.349639508 | 30S ribosomal protein S6 |
| EQ029_RS12205 | *rpmH* | 1.207259982 | 50S ribosomal protein L34 |
| Two-component system | | | |
| EQ029_RS00005 | *-* | -1.510935479 | chromosomal replication initiator protein DnaA |
| EQ029_RS00095 | *-* | 2.608742621 | response regulator transcription factor |
| EQ029_RS00100 | *walK* | 3.565512886 | cell wall metabolism sensor histidine kinase WalK |
| EQ029_RS00605 | *-* | -2.23484312 | M15 family metallopeptidase |
| EQ029_RS01570 | *-* | -1.13719023 | alkaline phosphatase |
| EQ029_RS02765 | *narJ* | 2.63500723 | nitrate reductase molybdenum cofactor assembly chaperone |
| EQ029_RS02775 | *nreA* | 1.859973909 | nitrate respiration regulation accessory nitrate sensor NreA |
| EQ029_RS02785 | *nreC* | 1.267812237 | nitrate respiration regulation response regulator NreC |
| EQ029_RS04440 | *-* | -1.792413921 | response regulator transcription factor |
| EQ029_RS04445 | *-* | -1.736049278 | GHKL domain-containing protein |
| EQ029_RS04455 | *-* | -1.250915083 | accessory gene regulator AgrB |
| EQ029_RS05750 | *-* | 1.022731334 | PAS domain-containing protein |
| EQ029_RS06985 | *-* | 1.520474995 | response regulator transcription factor |
| EQ029_RS06990 | *-* | 1.35041609 | GHKL domain-containing protein |
| EQ029_RS07240 | *mprF* | 1.693998027 | bifunctional lysylphosphatidylglycerol flippase/synthetase MprF |
| EQ029_RS07490 | *glnA* | 1.740564605 | type I glutamate--ammonia ligase |
| EQ029_RS08765 | *-* | -1.066754288 | cytochrome d ubiquinol oxidase subunit II |
| EQ029_RS08770 | *-* | -1.465614312 | cytochrome ubiquinol oxidase subunit I |
| EQ029_RS09470 | *dltD* | 4.176564821 | D-alanyl-lipoteichoic acid biosynthesis protein DltD |
| EQ029_RS09475 | *dltC* | 4.306326385 | D-alanine--poly(phosphoribitol) ligase subunit 2 |
| EQ029_RS09480 | *dltB* | 3.170562852 | D-alanyl-lipoteichoic acid biosynthesis protein DltB |
| EQ029_RS09485 | *dltA* | 3.56317078 | D-alanine--poly(phosphoribitol) ligase subunit DltA |
| EQ029_RS10355 | *-* | 1.375576995 | ABC transporter ATP-binding protein |
| EQ029_RS10360 | *-* | 1.429210154 | HAMP domain-containing histidine kinase |
| EQ029_RS10795 | *-* | -1.82963109 | acetyl-CoA C-acyltransferase |
| EQ029_RS12140 | *-* | 3.040703648 | ABC transporter ATP-binding protein |
| Glycolysis / Gluconeogenesis | | | |
| EQ029_RS00775 | *lpdA* | -1.982872861 | dihydrolipoyl dehydrogenase |
| EQ029_RS00790 | *-* | -1.910345552 | 2-oxo acid dehydrogenase subunit E2 |
| EQ029_RS01365 | *-* | -2.075630574 | PTS transporter subunit EIIC |
| EQ029_RS01450 | *-* | -2.227598627 | alcohol dehydrogenase catalytic domain-containing protein |
| EQ029_RS02125 | *-* | 1.484961706 | L-lactate dehydrogenase |
| EQ029_RS02225 | *-* | -1.902676538 | aldehyde dehydrogenase family protein |
| EQ029_RS03070 | *-* | 1.084914952 | galactose mutarotase |
| EQ029_RS04005 | *-* | 1.244713131 | fructose-bisphosphate aldolase |
| EQ029_RS04610 | *-* | 1.301912126 | aldehyde dehydrogenase |
| EQ029_RS06955 | *-* | 1.569598927 | PTS glucose transporter subunit IIA |
| EQ029_RS08710 | *lpdA* | -1.041045516 | dihydrolipoyl dehydrogenase |
| EQ029_RS08715 | *-* | -1.211956424 | 2-oxo acid dehydrogenase subunit E2 |
| EQ029_RS09330 | *-* | -1.554736846 | glucose-6-phosphate isomerase |
| EQ029_RS09755 | *eno* | -1.771958117 | phosphopyruvate hydratase |
| EQ029_RS09760 | *gpmI* | -3.324279826 | 2,3-bisphosphoglycerate-independent phosphoglycerate mutase |
| EQ029_RS09765 | *tpiA* | -2.129337179 | triose-phosphate isomerase |
| EQ029_RS09770 | *-* | -2.218652724 | phosphoglycerate kinase |
| EQ029_RS09775 | *gap* | -1.883578307 | type I glyceraldehyde-3-phosphate dehydrogenase |
| EQ029_RS11760 | *-* | 1.068434978 | histidine phosphatase family protein |
| EQ029_RS11840 | *-* | -2.477248689 | aldehyde dehydrogenase family protein |
| Quorum sensing | | | |
| EQ029_RS01080 | *-* | 2.275285272 | nickel ABC transporter substrate-binding protein |
| EQ029_RS01265 | *secA2* | -1.124989512 | accessory Sec system translocase SecA2 |
| EQ029_RS01445 | *-* | -1.999453788 | N-acyl homoserine lactonase family protein |
| EQ029_RS03570 | *secY* | 1.14715749 | preprotein translocase subunit SecY |
| EQ029_RS03665 | *lacD* | -4.578107101 | tagatose-bisphosphate aldolase |
| EQ029_RS04175 | *yidC* | -2.249346547 | membrane protein insertase YidC |
| EQ029_RS04440 | *-* | -1.792413921 | response regulator transcription factor |
| EQ029_RS04445 | *-* | -1.736049278 | GHKL domain-containing protein |
| EQ029_RS04455 | *-* | -1.250915083 | accessory gene regulator AgrB |
| EQ029_RS05345 | *ribD* | -3.722584202 | bifunctional diaminohydroxyphosphoribosylaminopyrimidine deaminase/5-amino-6-(5-phosphoribosylamino)uracil reductase RibD |
| EQ029_RS05975 | *yajC* | 2.520765469 | preprotein translocase subunit YajC |
| EQ029_RS07205 | *-* | -1.219796184 | anthranilate synthase component I |
| EQ029_RS09225 | *-* | 1.440344281 | peptide ABC transporter substrate-binding protein |
| EQ029_RS09230 | *-* | 1.805486037 | ABC transporter ATP-binding protein |
| EQ029_RS09235 | *-* | 2.002487424 | ABC transporter ATP-binding protein |
| EQ029_RS09240 | *-* | 2.145126034 | ABC transporter permease |
| EQ029_RS09245 | *-* | 1.908216 | ABC transporter permease |
| EQ029_RS09315 | *lepB* | 1.661024864 | signal peptidase I |
| EQ029_RS11040 | *secE* | 1.142035916 | preprotein translocase subunit SecE |
| EQ029_RS12130 | *lepB* | -1.73707283 | signal peptidase I |
| ABC transporters | | | |
| EQ029_RS00390 | *-* | 1.057461696 | metal ABC transporter  substrate-binding protein |
| EQ029_RS00400 | *-* | 1.472214983 | metal ABC transporter ATP-binding protein |
| EQ029_RS00560 | *rbsD* | -2.775499651 | D-ribose pyranase |
| EQ029_RS00565 | *-* | -2.487215983 | sugar ABC transporter ATP-binding protein |
| EQ029_RS00570 | *rbsC* | -1.738138116 | ribose ABC transporter permease |
| EQ029_RS00575 | *-* | -2.471192322 | D-ribose ABC transporter substrate-binding protein |
| EQ029_RS01205 | *-* | 1.112710446 | phosphate/phosphite/phosphonate ABC transporter substrate-binding protein |
| EQ029_RS01210 | *phnC* | 2.127554221 | phosphonate ABC transporter ATP-binding protein |
| EQ029_RS01215 | *phnE* | 1.345823721 | phosphonate ABC transporter, permease protein PhnE |
| EQ029_RS01335 | *-* | 3.80876682 | ABC transporter substrate-binding protein |
| EQ029_RS02245 | *-* | 1.28327101 | zinc ABC transporter substrate-binding protein |
| EQ029_RS02685 | *-* | 1.458179944 | amino acid ABC transporter permease |
| EQ029_RS02970 | *-* | 2.311220949 | FtsX-like permease family protein |
| EQ029_RS02975 | *-* | 1.480583663 | ABC transporter ATP-binding protein |
| EQ029_RS03310 | *-* | -3.435827147 | biotin transporter BioY |
| EQ029_RS03325 | *modA* | 1.100761179 | molybdate ABC transporter substrate-binding protein |
| EQ029_RS03330 | *modB* | 1.342376374 | molybdate ABC transporter permease subunit |
| EQ029_RS03740 | *-* | 2.051537392 | ABC transporter substrate-binding protein |
| EQ029_RS03875 | *-* | -2.981478807 | ATP-binding cassette domain-containing protein |
| EQ029_RS04880 | *-* | -1.005820197 | ABC transporter ATP-binding  protein |
| EQ029_RS07130 | *-* | 2.092381093 | phosphate ABC transporter ATP-binding protein |
| EQ029_RS07150 | *-* | 1.574646826 | ABC transporter substrate-binding protein |
| EQ029_RS08695 | *-* | -1.110739327 | ABC transporter ATP-binding protein |
| EQ029_RS09225 | *-* | 1.440344281 | peptide ABC transporter substrate-binding protein |
| EQ029_RS09230 | *-* | 1.805486037 | ABC transporter ATP-binding protein |
| EQ029_RS09235 | *-* | 2.002487424 | ABC transporter ATP-binding protein |
| EQ029_RS09240 | *-* | 2.145126034 | ABC transporter permease |
| EQ029_RS09245 | *-* | 1.908216 | ABC transporter permease |
| EQ029_RS09590 | *-* | 1.759896971 | MetQ/NlpA family ABC transporter substrate-binding protein |
| EQ029_RS09595 | *-* | 2.450103764 | ABC transporter permease |
| EQ029_RS09600 | *-* | 2.473839773 | methionine ABC transporter ATP-binding protein |
| EQ029_RS10055 | *-* | 1.837538185 | ABC transporter permease/substrate-binding protein |
| EQ029_RS10060 | *-* | 1.546741504 | ABC transporter ATP-binding protein |
| EQ029_RS10355 | *-* | 1.375576995 | ABC transporter ATP-binding protein |
| EQ029_RS10610 | *-* | 1.234934984 | ABC transporter substrate-binding protein |
| EQ029_RS12140 | *-* | 3.040703648 | ABC transporter ATP-binding protein |
| RNA degradation | | | |
| EQ029_RS04025 | *rho* | -1.099235283 | transcription termination factor Rho |
| EQ029_RS04230 | *-* | 1.020788581 | DEAD/DEAH box helicase |
| EQ029_RS04485 | *groL* | -4.028034844 | chaperonin GroEL |
| EQ029_RS06230 | *dnaK* | -5.309516875 | molecular chaperone DnaK |
| EQ029_RS07605 | *rny* | -2.631161858 | ribonuclease Y |
| EQ029_RS09735 | *rnr* | -1.235748614 | ribonuclease R |
| EQ029_RS09755 | *eno* | -1.771958117 | phosphopyruvate hydratase |
| EQ029_RS10065 | *recQ* | -1.091543033 | DNA helicase RecQ |

**C :** 16 μg/mL VS 8 μg/mL

| Gene ID | Name | log2FC | Description |
| --- | --- | --- | --- |
| Ribosome | | | |
| EQ029_RS00070 | *rplI* | -1.135253341 | 50S ribosomal protein L9 |
| EQ029_RS03465 | *rpsJ* | 4.072655224 | 30S ribosomal protein S10 |
| EQ029_RS03470 | *rplC* | 1.446563324 | 50S ribosomal protein L3 |
| EQ029_RS03475 | *rplD* | 2.716910955 | 50S ribosomal protein L4 |
| EQ029_RS03480 | *rplW* | 2.591835243 | 50S ribosomal protein L23 |
| EQ029_RS03485 | *rplB* | 3.657941798 | 50S ribosomal protein L2 |
| EQ029_RS03490 | *rpsS* | 1.376726045 | 30S ribosomal protein S19 |
| EQ029_RS03495 | *rplV* | 3.738762504 | 50S ribosomal protein L22 |
| EQ029_RS03500 | *rpsC* | 2.52342359 | 30S ribosomal protein S3 |
| EQ029_RS03505 | *rplP* | 3.123761721 | 50S ribosomal protein L16 |
| EQ029_RS03510 | *rpmC* | 4.40612143 | 50S ribosomal protein L29 |
| EQ029_RS03515 | *rpsQ* | 1.485433053 | 30S ribosomal protein S17 |
| EQ029_RS03525 | *rplX* | 1.25268204 | 50S ribosomal protein L24 |
| EQ029_RS03530 | *rplE* | 2.63261453 | 50S ribosomal protein L5 |
| EQ029_RS03535 | *-* | 4.869854258 | type Z 30S ribosomal protein S14 |
| EQ029_RS03540 | *rpsH* | 1.957951693 | 30S ribosomal protein S8 |
| EQ029_RS03545 | *rplF* | 2.341329451 | 50S ribosomal protein L6 |
| EQ029_RS03550 | *rplR* | 3.201813883 | 50S ribosomal protein L18 |
| EQ029_RS03555 | *rpsE* | 1.631532881 | 30S ribosomal protein S5 |
| EQ029_RS03560 | *rpmD* | 1.276372857 | 50S ribosomal protein L30 |
| EQ029_RS03565 | *rplO* | 1.386092654 | 50S ribosomal protein L15 |
| EQ029_RS03590 | *rpsM* | 1.296464379 | 30S ribosomal protein S13 |
| EQ029_RS03595 | *rpsK* | 2.390694336 | 30S ribosomal protein S11 |
| EQ029_RS03630 | *rplM* | 1.632855681 | 50S ribosomal protein L13 |
| EQ029_RS03635 | *rpsI* | 3.409058089 | 30S ribosomal protein S9 |
| EQ029_RS04030 | *-* | 3.763656517 | type B 50S ribosomal protein L31 |
| EQ029_RS05600 | *rpsD* | 3.986906484 | 30S ribosomal protein S4 |
| EQ029_RS05805 | *rpmI* | 3.580927578 | 50S ribosomal protein L35 |
| EQ029_RS05810 | *rplT* | 4.134311492 | 50S ribosomal protein L20 |
| EQ029_RS06205 | *rpsT* | 3.256264934 | 30S ribosomal protein S20 |
| EQ029_RS06255 | *rpsU* | -2.314919829 | 30S ribosomal protein S21 |
| EQ029_RS06375 | *rpmG* | 1.122349521 | 50S ribosomal protein L33 |
| EQ029_RS07355 | *rpmG* | 2.391702788 | 50S ribosomal protein L33 |
| EQ029_RS07670 | *rpsO* | 2.507810341 | 30S ribosomal protein S15 |
| EQ029_RS07755 | *rpsB* | 2.872616882 | 30S ribosomal protein S2 |
| EQ029_RS07830 | *rplS* | 1.994971537 | 50S ribosomal protein L19 |
| EQ029_RS07845 | *rpsP* | 3.845760334 | 30S ribosomal protein S16 |
| EQ029_RS10980 | *rpsG* | 1.926242752 | 30S ribosomal protein S7 |
| EQ029_RS10985 | *rpsL* | 2.357040034 | 30S ribosomal protein S12 |
| EQ029_RS11010 | *rplL* | 4.250334488 | 50S ribosomal protein L7/L12 |
| EQ029_RS11015 | *-* | 4.93155029 | 50S ribosomal protein L10 |
| EQ029_RS11025 | *rplA* | 2.826101106 | 50S ribosomal protein L1 |
| EQ029_RS11030 | *rplK* | 1.716168191 | 50S ribosomal protein L11 |
| EQ029_RS11045 | *rpmG* | -1.359908157 | 50S ribosomal protein L33 |
| EQ029_RS11300 | *-* | 3.113012699 | 50S ribosomal protein L25/general stress protein Ctc |
| Two-component system | | | |
| EQ029_RS00095 | *-* | 2.187220733 | response regulator transcription factor |
| EQ029_RS00100 | *walK* | 3.114897449 | cell wall metabolism sensor histidine kinase WalK |
| EQ029_RS00605 | *-* | -1.242066809 | M15 family metallopeptidase |
| EQ029_RS02755 | *-* | -2.78467784 | nitrate reductase subunit alpha |
| EQ029_RS02760 | *narH* | -1.662643571 | nitrate reductase subunit beta |
| EQ029_RS02770 | *narI* | -1.598409226 | respiratory nitrate reductase subunit gamma |
| EQ029_RS02780 | *nreB* | -1.0234191 | nitrate respiration regulation sensor histidine kinase NreB |
| EQ029_RS02790 | *-* | -2.396666077 | NarK/NasA family nitrate transporter |
| EQ029_RS04440 | *-* | -1.960748667 | response regulator transcription factor |
| EQ029_RS04445 | *-* | -2.31159974 | GHKL domain-containing protein |
| EQ029_RS04455 | *-* | -2.724318317 | accessory gene regulator AgrB |
| EQ029_RS04780 | *-* | 2.388014229 | sensor histidine kinase |
| EQ029_RS04785 | *vraR* | 1.226811297 | two-component system response regulator VraR |
| EQ029_RS05695 | *-* | 1.052746587 | NAD-dependent malic enzyme 4 |
| EQ029_RS05750 | *-* | 1.359083497 | PAS domain-containing protein |
| EQ029_RS07240 | *mprF* | 1.831198322 | bifunctional lysylphosphatidylglycerol flippase/synthetase MprF |
| EQ029_RS07430 | *-* | -1.738381516 | sensor histidine kinase |
| EQ029_RS07490 | *glnA* | 1.533544574 | type I glutamate--ammonia ligase |
| EQ029_RS08610 | *-* | -1.300717318 | heme A synthase |
| EQ029_RS09470 | *dltD* | 3.877123575 | D-alanyl-lipoteichoic acid biosynthesis protein DltD |
| EQ029_RS09475 | *dltC* | 3.685877744 | D-alanine--poly(phosphoribitol) ligase subunit 2 |
| EQ029_RS09480 | *dltB* | 3.438960513 | D-alanyl-lipoteichoic acid biosynthesis protein DltB |
| EQ029_RS09485 | *dltA* | 3.367830104 | D-alanine--poly(phosphoribitol) ligase subunit DltA |
| EQ029_RS10795 | *-* | -3.244457305 | acetyl-CoA C-acyltransferase |
| EQ029_RS12140 | *-* | 2.127817076 | ABC transporter ATP-binding protein |
| Quorum sensing | | | |
| EQ029_RS01080 | *-* | 1.636274924 | nickel ABC transporter substrate-binding protein |
| EQ029_RS01265 | *secA2* | -1.383161969 | accessory Sec system translocase SecA2 |
| EQ029_RS01445 | *-* | -3.438879653 | N-acyl homoserine lactonase family protein |
| EQ029_RS02580 | *yidC* | -1.257603227 | membrane protein insertase YidC |
| EQ029_RS03570 | *secY* | 1.478937723 | preprotein translocase subunit SecY |
| EQ029_RS03665 | *lacD* | -2.201261146 | tagatose-bisphosphate aldolase |
| EQ029_RS03960 | *-* | 1.832380294 | S-ribosylhomocysteine lyase |
| EQ029_RS04175 | *yidC* | -1.114976157 | membrane protein insertase YidC |
| EQ029_RS04440 | *-* | -1.960748667 | response regulator transcription factor |
| EQ029_RS04445 | *-* | -2.31159974 | GHKL domain-containing protein |
| EQ029_RS04455 | *-* | -2.724318317 | accessory gene regulator AgrB |
| EQ029_RS05345 | *ribD* | -3.228509988 | bifunctional diaminohydroxyphosphoribosylaminopyrimidine deaminase/5-amino-6-(5-phosphoribosylamino)uracil reductase RibD |
| EQ029_RS05375 | *-* | -3.08714074 | MarR family transcriptional regulator |
| EQ029_RS05975 | *yajC* | 2.526999821 | preprotein translocase subunit YajC |
| EQ029_RS05980 | *secDF* | 1.19940497 | protein translocase subunit SecDF |
| EQ029_RS07515 | *hfq* | 1.334647056 | RNA chaperone Hfq |
| EQ029_RS09025 | *-* | 1.412653722 | competence protein ComK |
| EQ029_RS09230 | *-* | 1.213200928 | ABC transporter ATP-binding protein |
| EQ029_RS09235 | *-* | 1.688177734 | ABC transporter ATP-binding protein |
| EQ029_RS09240 | *-* | 1.997169279 | ABC transporter permease |
| EQ029_RS09245 | *-* | 1.548698091 | ABC transporter permease |
| EQ029_RS09315 | *lepB* | 2.799258827 | signal peptidase I |
| EQ029_RS09320 | *lepB* | 2.052831197 | signal peptidase I |
| EQ029_RS11040 | *secE* | -1.045401523 | preprotein translocase subunit SecE |
| EQ029_RS12130 | *lepB* | -2.065549843 | signal peptidase I |
| ABC transporters | | | |
| EQ029_RS00560 | *rbsD* | -1.577303473 | D-ribose pyranase |
| EQ029_RS00570 | *rbsC* | -0.085059127 | ribose ABC transporter permease |
| EQ029_RS03325 | *modA* | -1.128915676 | molybdate ABC transporter substrate-binding protein |
| RNA degradation | | | |
| EQ029_RS04025 | *rho* | -0.040283254 | transcription termination factor Rho |
| EQ029_RS06230 | *dnaK* | -3.778529949 | molecular chaperone DnaK |
| EQ029_RS09755 | *eno* | 0.11126323 | phosphopyruvate hydratase |

**Supplementary Table 5**

| Gene ID | Name | log2FC | Description |
| --- | --- | --- | --- |
| EQ029_RS09810 | *clpP* | -3.388854301 | ATP-dependent Clp endopeptidase proteolytic subunit ClpP |
| EQ029_RS05830 | *clpX* | -0.591046198 | ATP-dependent Clp protease ATP-binding subunit ClpX |
| EQ029_RS09270 | *clpB* | -4.176444959 | ATP-dependent chaperone ClpB |
| EQ029_RS04480 | *groES* | -4.059160722 | co-chaperone GroES |
| EQ029_RS04485 | *groL* | -4.028034844 | chaperonin GroEL |
| EQ029_RS06225 | *grpE* | -5.226939471 | nucleotide exchange factor GrpE |
| EQ029_RS06235 | *dnaJ* | -5.838062743 | molecular chaperone DnaJ |
| EQ029_RS11745 | *ahpC* | -0.032740887 | peroxiredoxin |
| EQ029_RS00965 | *bioB* | -5.98515388 | biotin synthase BioB |
